# Descriptive and Multivariate Analysis of the Pig Sector in North Macedonia and Its Implications for African Swine Fever Transmission

**DOI:** 10.3389/fvets.2021.733157

**Published:** 2021-11-30

**Authors:** Kathleen C. O'Hara, Daniel Beltrán-Alcrudo, Mark Hovari, Blagojco Tabakovski, Beatriz Martínez-López

**Affiliations:** ^1^Center for Animal Disease Modeling and Surveillance (CADMS), School of Veterinary Medicine, University of California, Davis, Davis, CA, United States; ^2^Food and Agriculture Organization of the United Nations (FAO) Regional Office for Europe and Central Asia, Budapest, Hungary; ^3^Food Veterinary Administration, Skopje, Macedonia

**Keywords:** African swine fever, biosecurity risk score, kernel density estimation, multiple correspondence analysis, North Macedonia, Kosovo

## Abstract

North Macedonia, a country in the Balkan region of Europe, is currently bordered to the north and east by countries with active African swine fever (ASF) outbreaks. The predominantly traditional backyard pig farming sector in this country is under imminent threat of disease incursion. The characteristics and practices of such sectors have rarely been described, and thus the implications for these factors on disease introduction and spread are poorly understood. Using a semi-structured questionnaire, 457 pig producers were interviewed, providing information on 77.7% of the pig population in North Macedonia. In addition, a pilot study of 25 pig producers in Kosovo was performed. This study aimed to provide a detailed description of the North Macedonian pig sector, to make comparisons with nearby Kosovo, and to identify areas with high-risk practices for targeted mitigation. Descriptive data were summarized. Results of the questionnaire were used to identify farm-level risk factors for disease introduction. These factors were used in the calculation of a biosecurity risk score. Kernel density estimation methods were used to generate density maps highlighting areas where the risk of disease introduction was particularly concentrated. Multiple correspondence analysis with hierarchical clustering on principal components was used to explore patterns in farm practices. Results show that farms were predominantly small-scale with high rates of turnover. Pig movement was predominantly local. The highest biosecurity risk scores were localized in the eastern regions of North Macedonia, concerningly the same regions with the highest frequency of wild boar sightings. Veterinarians were highly regarded, regularly utilized, and trusted sources of information. Practices that should be targeted for improvement include isolation of new pigs, and consistent application of basic sanitary practices including washing hands, use of disinfection mats, and separation of clean and dirty areas. This study provides the most complete description of the North Macedonian pig sector currently available. It also identifies regions and practices that could be targeted to mitigate the risk of disease incursion and spread. These results represent the first steps to quantify biosecurity gaps and high-risk behaviors in North Macedonia, providing baseline information to design risk-based, more cost-effective, prevention, surveillance, and control strategies.

## Introduction

The Republic of North Macedonia is located on the Balkan Peninsula in Southeast Europe. It is bordered by Kosovo^1^ and Serbia to the north, Bulgaria to the east, Greece to the south, and Albania to the west. Bulgaria and Serbia are currently experiencing outbreaks of African swine fever (ASF) in both domestic pigs and wild boar, while Greece reported a single introduction in domestic pigs in 2020. African swine fever is a World Organization for Animal Health (OIE) reportable, viral haemorrhagic disease of domestic and wild suids (1). Depending on the viral strain and host factors, ASF infection can present as peracute, acute, subacute, or chronic disease. The virus circulating in the Balkans (and the rest of Europe except for the Italian island of Sardinia, plus in Asia) is of genotype II and acute or peracute in its clinical presentation (among others, genotype II is also present in Africa) (1, 2). Peracute cases are rapidly progressive, presenting with high fever, lethargy, anorexia and/or sudden death. Acute cases may be characterized by high fever, depression, anorexia, vomiting, diarrhea, abortion, haemorrhagic lesions and/or sudden death; while subacute or chronic cases may range from inapparent to having intermittent fevers, lethargy, weight loss, skin ulcers, arthritis and/or respiratory signs (3, 4). When introduced to naïve populations, ASF can result in up to 100% lethality if no mitigation is enacted (4, 5). Wild boar and domestic pigs are equally affected by the disease. Wild boar are of concern due to their contribution to the maintenance and spread of this disease in Europe; while warthogs and likely bushpigs are asymptomatic and contribute to the sylvactic cycle in Africa together with soft ticks of the genus *Ornithodoros* (6–12). Disease transmission in both domestic and wild pigs can occur via direct contact with an infected animal, consumption of contaminated materials (e.g., swill feeding, discarded offal, scavenged carcasses or garbage), exposure to fomites, iatrogenically, or through the bite of infected *Ornithodoros* ticks if present in the area (7, 8, 13–17). No treatment and no vaccines currently exist for ASF. Control is dependent on strict biosecurity, surveillance, rapid detection and stamping out with compensation (5, 12, 14, 18, 19). The absence of a vaccine and the survival of the virus in ticks and the wild pig population, make full eradication after introduction is challenging, with few examples in recent years, namely Belgium, Czech Republic, and Greece (20). The introduction of ASF into a disease-free country can result in massive economic impacts via direct losses to the disease (i.e., mortality, stamping out, control measures etc.) or secondary losses associated with trade restrictions (21). In Europe, trade losses have greatly surpassed direct losses for countries exporting pigs and pork products. Control measures have been associated with high costs due to stamping out of infected farms. Within the Balkan region, ASF was first reported in Bulgaria in August 2018, in Serbia in August 2019, and in Greece in February 2020 (1). While Greece's only outbreak affected domestic pigs, Bulgaria and Serbia's outbreaks have impacted both domestic pig and wild boar populations (1). With this rapid timeline, the surrounding active outbreaks, and the mobility of infected wild boar, the pig industries in North Macedonia and Kosovo, while currently free of African swine fever, are under imminent threat of disease incursion.

Within North Macedonia, the Food and Veterinary Agency (FVA) developed programs and policies, and distributed educational materials, to aid in the prevention of ASF introduction into the country and to improve early detection efforts. The FVA had a full ASF awareness campaign starting in 2018, which included billboards and leaflets, and media releases via radio and television. With the support of the Food and Agriculture Organization of the United Nations (FAO), the following awareness and training efforts were implemented: (1) the distribution to field veterinarians of several hundreds of the FAO manual on ASF detection and diagnosis in Macedonian; (2) ongoing distribution of editable ASF leaflets; (3) four veterinarians attended a training-of-trainers event in September 2019; (4) 10 official veterinarians and 15 private veterinarians attended a biosecurity workshop in October 2019, (5) an ASF outbreak simulation exercise for official veterinarians was run in November 2019, and (6) a 4-week online certified training on ASF preparedness in Serbian. Additionally, FAO, in collaboration with the Veterinary Chamber of the Republic of North Macedonia (a non-profit organization of veterinarians and the veterinary statutory body for the country), undertook a survey of the pig industry to better characterize and define current husbandry practices, socioeconomic aspects, biosecurity capabilities, and disease awareness. FAO also administered this questionnaire to a small sample of pig farmers in Kosovo. This report will present the findings of this collaborative effort and provide some initial targets for ongoing mitigation efforts.

## Materials and Methods

A questionnaire was designed and implemented by FAO to gather information about husbandry, veterinary care, socioeconomics, the pork value chain, biosecurity, and disease awareness throughout the pig sector in North Macedonia and Kosovo. The questionnaires were adapted from earlier work conducted by FAO in Georgia (22, 23). FAO followed the principles of the declaration of Helsinki and the Belmont report when designing and implementing the survey. The Institutional Review Board (IRB) of UC Davis Administration issued an exemption from the requirement for IRB review, the reasons being that the surveys would not elicit responses that would place the respondents at risk if obtained by individuals not associated with the research. The exemption criteria are available at 45 CFR 46.101(b)(2)–U.S. Code of Federal Regulation, Protection of human subjects. All the interviewed producers were informed of the study purpose, and of the facts that participation in the interviews was voluntary and they could drop from the study at any time.

### Questionnaire

Semi-structured questionnaires were originally written in English and subsequently translated into Macedonian. In Kosovo, questionnaires were presented in English and translated into Serbian and Albanian by the surveyor as needed. Questionnaires included sections on: husbandry, veterinary care, socioeconomics, pork value chain, biosecurity including cleaning protocols, visitor access, exposure to other domestic and wild pigs, swill feeding practices and waste management and ASF awareness (Appendix 1). All questions referred to the 12 months prior to the date of interview. Questions related to slaughter focused on homeslaughter practices. North Macedonia has 14 commercial slaughterplants that process multiple species; however, these were not captured in the survey.

### Sample Selection

#### North Macedonia

Pig holdings, as identified by an annual census, were divided into three groups based on the number of pigs present: >100 commercial, 11–100 family farm, and 0–10 backyard farm. Based on the 2019 pig census, the pig population of North Macedonia consists of around 125,230 pigs, distributed across 2,315 farms with an average of 58 animals per farm. Under EU legislation, holdings with one pig for domestic purposes are not required to register, therefore these farms may be underrepresented in this count; illegal holdings are not thought to be an issue in North Macedonia. Five hundred farms were targeted, including all commercial farms (*n* = 77), and a 2:1 split of family (*n* = 282) and backyard (*n* = 141) farms focusing on those farms with the most pigs. North Macedonia is divided into progressively smaller administrative levels: regions, municipalities, and town/villages, respectively. Family and backyard farms were proportionally divided between regions (but not municipalities). Within regions, and taking into account the availability of private veterinarians, farms were randomly selected for interviews. These farms were then visited to administer the questionnaires in person.

#### Kosovo

In Kosovo the major distinction was made between commercial (> 100 animals) and non-commercial farms (≤100 animals). The pig population of Kosovo consists of around 42,000 pigs distributed between one commercial farm and 3,948 non-commercial farms with an average of 11 animals per farm. Twenty-five farms were surveyed during a pilot study in August-September 2020. One survey was carried out in the one commercial farm in Kosovo located in Viti, while the remaining 24 samples were divided evenly into 12 surveys from the Serbian speaking community in the North and 12 samples from the Catholic Albanian community in the West. Farms were selected based on convenience and recommendations of the local veterinary offices.

### Data Collection

#### North Macedonia

In North Macedonia, questionnaires were conducted through the Veterinary Chamber of North Macedonia by private veterinarians selected based on the villages and municipalities they served. Prior to questionnaire implementation, training sessions were organized in each region for the interviewers, covering the survey goals, content, schedule, and basic interview techniques. Survey data was collected via the Epicollect5 mobile platform (24). Interviews were conducted between September 2019 and March 2020. A total of 457 questionnaires were implemented and are analyzed here. The semi-structured format of the survey allowed respondents to select multiple responses for some questions, therefore percentages discussed below represent the percent of respondents selecting a given answer—a given respondent may be counted across multiple answers if they selected more than one response.

#### Kosovo

In Kosovo one surveyor was hired and trained to fill in the twenty-five surveys in all of the locations. Data collection was also done via Epicollect5.

#### Data Definitions

When collecting information on the types of pigs, sows were defined as females with litters in the last 12 months. The total number of pigs per farm was calculated as the sum of the reported boars, fattening pigs, piglets, and sows.

### Data Analysis

Descriptive statistics were computed from the questionnaire results from North Macedonia and Kosovo. Summary information on husbandry, veterinary care and practices, the pork value chain, biosecurity, and disease awareness, is presented as the proportion of respondents selecting or providing given answers (Appendix 2). Multiple choice questions allowed respondents to select multiple answers, meaning that one producer's response may contribute to the proportion of respondents for multiple answers. Data processing and analyses were performed in R Studio (v3.6.1) (25). Spatial visualization and analyses were performed in ArcGIS Desktop v10.7. Mapping was conducting using the World Azimuthal Equidistant Projection.

#### Biosecurity Risk Scores

Biosecurity risk scores were calculated for farms in North Macedonia using a subset of responses from the questionnaire. Based on established literature and subject matter expertise, risk factors for disease introduction were identified and 28 questions that reflect those factors were selected: 21 questions that were answered by all farms, and an additional seven questions that were answered by family and commercial farms only. The answers to each of these questions were dichotomized, such that high risk answers/behaviors were assigned a score of one, and no/low risk answers/behaviors were assigned a score of zero (Supplementary Table 1). Missing values were scored as zero. A biosecurity risk score was calculated as a non-weighted linear combination of these values for each farm. The higher the biosecurity risk score, the worse the biosecurity practices were on that farm (maximum score for all farms: 21, maximum score for family and commercial farms: 28). Biosecurity risk scores were calculated for North Macedonia; due to limited data biosecurity risk scores were not calculated for Kosovo.

#### Generation of Highest Biosecurity Risk Maps Using Kernel Density Estimation

Kernel density estimation (KDE) is a non-parametric method to estimate the probability density function of a variable (26). Using our biosecurity risk score, each farm serves as a point over which KDE fits a smooth curve with the true value at the exact location of the farm and diminishing values estimated with increasing distance from the farm/known biosecurity risk score. Using this method, we generated maps estimating the areas with highest biosecurity risk based on biosecurity risk scores from all farms. Additionally, we also generated risk maps using the biosecurity risk scores from family and commercial farms who answered both the initial 21 questions and the additional subset of seven biosecurity questions. KDE was used to generate risk maps for North Macedonia; risk maps were not generated for Kosovo due to the limited amount of data available. The kernel density function within ArcGIS was used, specifying a search radius of 10 Km and an output cell size of 1 Km.

#### Generation of Farm Profiles Using Multiple Correspondence Analysis With Hierarchical Clustering on Principal Components

Multiple correspondence analysis (MCA) is an extension of simple correspondence analysis used for analyzing the association between two or more qualitative variables (27–29). MCA is able to take the many variables generated by our survey responses and evaluate how they may be associated, e.g., if a respondent selected a specific answer to one question, is that associated with answering another question in a certain way? MCA further allows us to visualize the associations between variables by plotting them in space; variables near each other share a similar profile.

MCA was performed via forward stepwise selection selecting for the highest level of variance explained, resulting in the inclusion of nine categorical variables: household income from pigs, fate of meat and pork products produced, do you wash hands before going to pigs, do you use disinfection mat before going to pigs, which people are allowed access to your pigs, do you bring in external boar for mating purposes, biosecurity risk score, farm type and region. Farm type and region were used as supplemental variables, meaning they did not contribute to the calculation of the principle dimensions, but their coordinates were predicted to estimate how they might relate to those variables included in the analysis. Household income derived from pig production was divided into a categorical variable of ≤50%, or >50%. Fate of products was divided into slaughtered for home consumption vs. slaughtered for any other purpose. People pig access was divided into no access, veterinarians, and any other combination. External boar was divided into those farms that allowed their animals to interact with other pigs (their boar goes offsite, sows are crossed offsite, or external boar come to their farm), and those that allowed no interaction with other pigs. Biosecurity risk score was divided into low (0–2; lowest 50%), medium (3–5; middle 51–89%) or high (≥6; top 10%) risk.

After the MCA, we used hierarchical clustering on principle components (HCPC), which is a methodology that clusters individuals according to similar patterns of variable responses, e.g., two respondents who had similar answer profiles would be grouped together (30). HCPC grouped farms based on similar patterns in their survey responses. This allowed us to generate biosecurity farm profiles or groups of farms that share specific farm characteristics as defined by their questionnaire responses. MCA and HCPC were performed in R Studio using the FactoMineR (31) and factoextra (32) packages. HCPC was performed using Ward's criteria. The number of clusters was determined using the “elbow method,” which entails plotting the explained variation as a function of the number of clusters and selecting the elbow of the curve as the best balance between number of clusters and variance explained (32).

## Results

**Figure 1:**
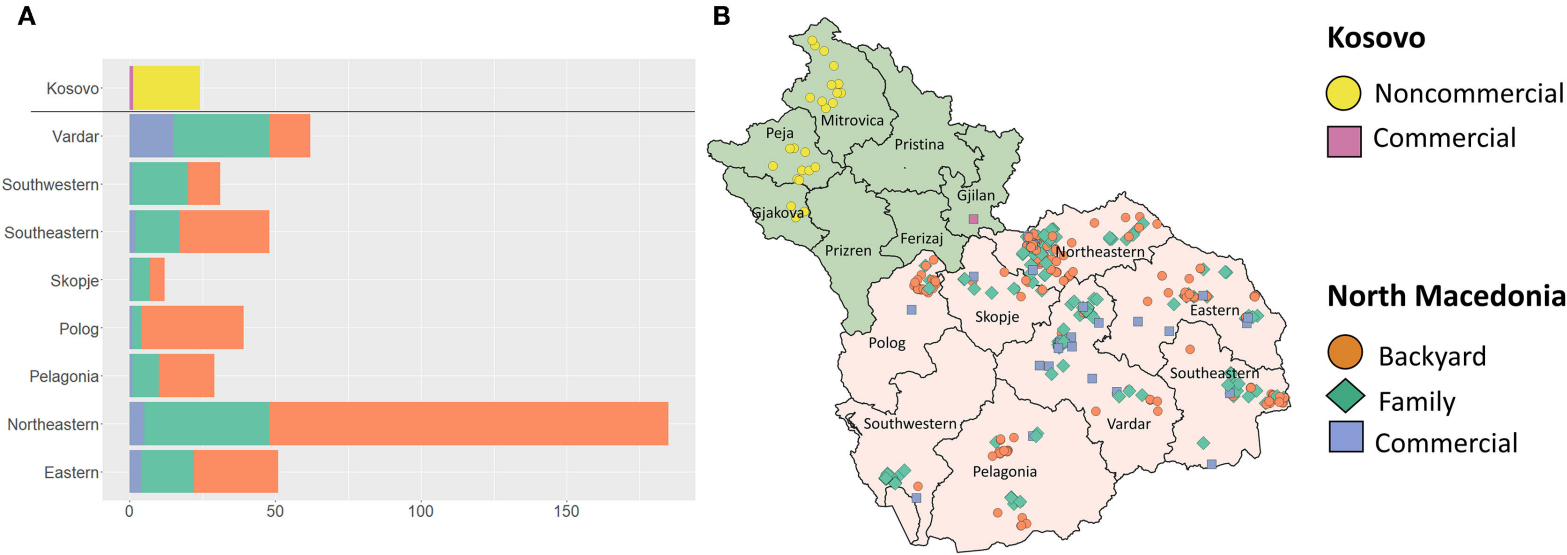
**(A)** Number of questionnaires administered to North Macedonian pig producers by region and type of farm, and Kosovar producers characterized as commercial or non-commercial, during September 2019-March 2020. **(B)** Map of questionnaire sites by farm type. Kosovo: green, North Macedonia: orange. Kosovo districts and North Macedonia Regions: black lines.

A total of 457 surveys were completed in North Macedonia by March 29, 2020 (251 in 2019, 206 in 2020); 281 backyard (61.5% of respondents), 146 family (31.9% of respondents) and 30 commercial (6.6% of respondents) farms. The surveyed farms accounted for 77.7% of the pig population in North Macedonia. Additionally, a total of 25 questionnaires were administered during a pilot study in Kosovo, representing 24 non-commercial farms (≤100 pigs) and one commercial farm (>100 pigs). The breakdown of surveys by farm type and region/district are presented in Figure 1.

### Husbandry

The number of sows, boars, fattening pigs, and piglets reported on North Macedonian farms was assessed by farm type (Table 1). Producers were asked about the current number of pigs, as well as the minimum and maximum numbers of each type of pig present on-site in the last 12 months (Table 1). Backyard and family farms tended to have more piglets than fattening pigs, in contrast to commercial farms in which fattening pigs predominate (Table 1). Overall, across pig and farm types, the number of pigs on any individual farm changed by about 30% over the course of a year. Commercial farms had more stable pig numbers, changing by 20–30%, compared to backyard or family farms whose pig numbers may change by up to 50–60%; fattening pigs and piglets had the highest turnover.

**Table 1:** Number of pigs by farm type as reported by questionnaires administered to North Macedonian pig producers between September 2019 and March 2020.

**Farm types**		**Sows**	**Boars**	**Fattening pigs**	**Piglets**	**Total**
All	Mean (SD)	7 (53)	1 (2)	121 (771)	84 (501)	213 (1,304)
	Median	1	0	2	6	11
	Avg minimum (SD)	8 (52)	1 (2)	97 (644)	66 (452)	
	Avg maximum (SD)	11 (59)	1 (3)	145 (866)	91(555)	
	%Change AvgMax-AvgMin	0.3	0.3	0.3	0.3	
Backyard	Mean (SD)	1 (4)	0 (1)	3 (7)	7 (12)	11 (18)
	Median	1	0	1	2	6
	Avg minimum (SD)	2 (2)	0 (1)	2 (3)	7 (12)	
	Avg maximum (SD)	3 (4)	0 (1)	5 (13)	11 (18)	
	%Change AvgMax-AvgMin	0.4	0.2	0.6	0.4	
Family	Mean (SD)	3 (5)	1 (2)	30 (64)	35 (54)	69 (112)
	Median	2	1	3	20	29
	Avg minimum (SD)	5 (7)	1 (1)	16 (37)	23 (46)	
	Avg maximum (SD)	9 (8)	1 (4)	43 (77)	48 (86)	
	%Change AvgMax-AvgMin	0.5	0.4	0.6	0.5	
Commercial	Mean (SD)	82 (195)	5 (6)	1,669 (2,584)	1,043 (1,707)	2,799 (4,386)
	Median	15	2	460	335	737
	Avg minimum (SD)	82 (189)	4 (5)	1,371 (2,171)	830 (1,597)	
	Avg maximum (SD)	103 (210)	6 (8)	1,945 (2,858)	1,047 (1,947)	
	%Change AvgMax-AvgMin	0.2	0.3	0.3	0.2	

*The number of pigs currently on the farm were reported by type of pig. Producers also separately reported the maximum and minimum number of each type of pig that were on the farm in the last 12 months. Total pigs were calculated as the sum of the reported sows, boars, fattening pigs and piglets currently on-site. Percent change in average number of pigs was calculated as the difference between the average maximum and average minimum divided by the average maximum*.

*SD, standard deviation; Avg, average; Avg Minimum, average of the minimum number of each type of pig reported; Avg Maximum, average of the maximum number of each type of pig reported; %Change, percent change*.

In North Macedonia, commercial breeds of pigs were the most common, with 96.7% of commercial farms, 65.8% of family farms, and 76.1% of backyard farms reporting only commercial breeds; the remainder reported local breeds only (commercial 0.0%, family 31.5%, backyard 22.4%), or a combination of local and commercial breeds (commercial 3.3%, family 2.7%, backyard 3.2%). In Kosovo, half of respondents reported only local breeds (48.0%), while the other half reported a combination of local and commercial breeds (48.0%); 4.0% reported commercial breeds only.

In North Macedonia, commercial operations used the highest proportion of hired workers to take care of their pigs (80.0%). Among backyard and family farms, husbands (83.8%) and wives (50.8%) were the most common pig caretakers, with children (21.5%), other family (15.9%), and rarely hired workers (2.8%) also contributing. More Kosovar respondents reported wives (80%) and kids (44%) caring for pigs, in addition to husbands (100%).

**Figure 2:**
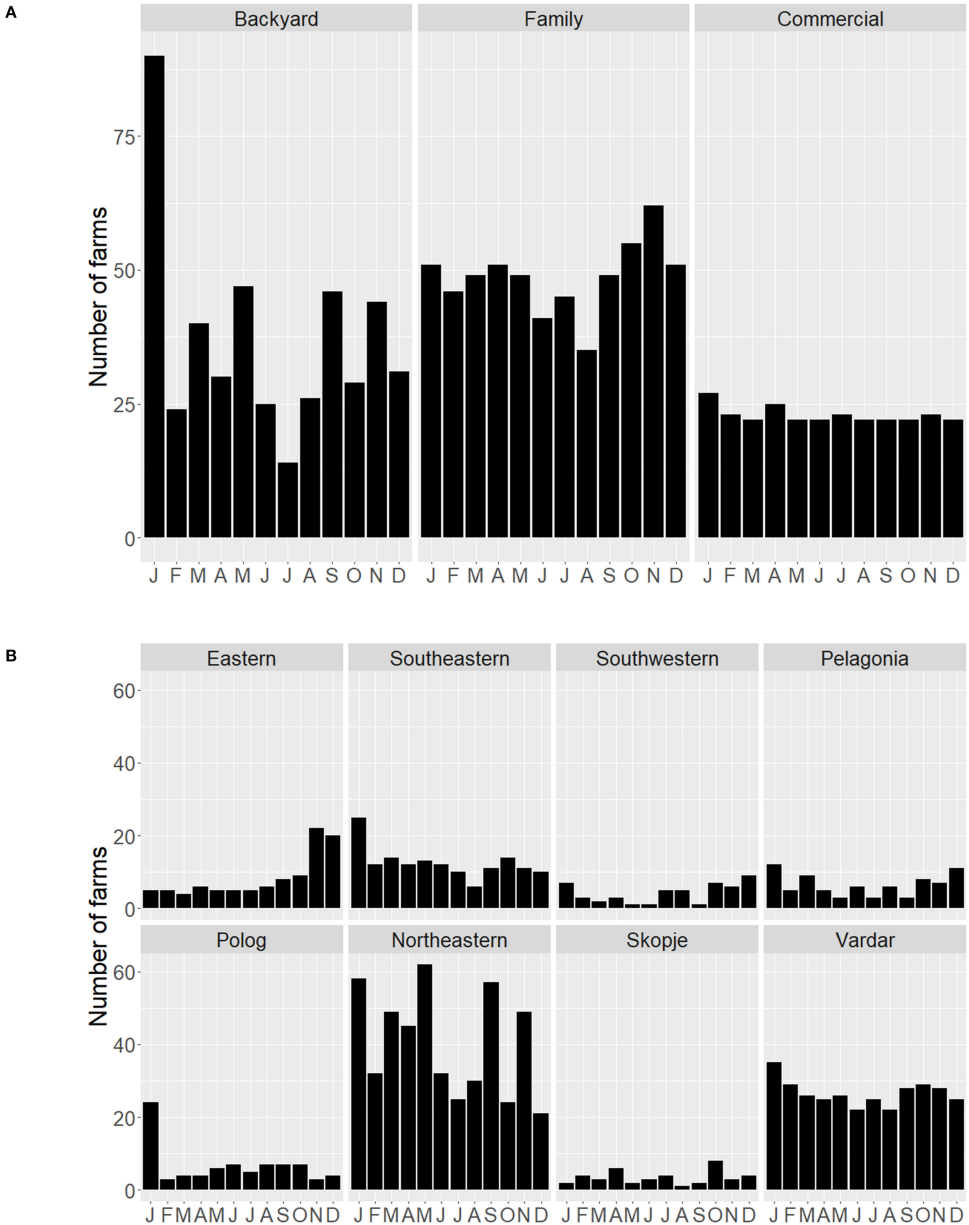
Number of North Macedonian pig farms reporting litters per month by **(A)** farm type, and **(B)** region, based on questionnaires administered between September 2019 and March 2020.

In North Macedonia, among backyard and family farms, the births of pig litters were seasonal; both farm types reported fewer litters over summer, with peaks in spring and winter (Figure 2A). Commercial farms reported litters being delivered throughout the year. The spring peak observed for backyard and family farms was variable by region, being most pronounced in Pelagonia, Northeastern, and Skopje (Figure 2B). Within Kosovo, births were concentrated in the spring, with the commercial farm reporting year-round litters.

North Macedonian pigs were predominately fed with grain (97.2%) and commercial feed (38.7%); commercial farms reported they only feed grain and commercial feed. About 15.1% of North Macedonian farms fed grass. Hay (7.2%) and agricultural by-products (6.6%) were each used to a lesser extent than other feed items. Butcher waste and food processing by-products were used by <1.0% of producers in North Macedonia. Food scraps were fed by 6.8% of farms in North Macedonia. Ninety-four percent of North Macedonian farms feeding food scraps reported the scraps they fed were from their own household. In North Macedonia, one backyard farm reported feeding scraps from a restaurant and one from a market. Of those North Macedonian farms feeding food scraps, 56.8% reported that they boil the scraps before feeding them to pigs. Only 3.5% of North Macedonian respondents report that their pigs were allowed to scavenge (during the day, returning at night), with the remainder keeping their pigs enclosed year-round. Three of these farms explicitly report allowing scavenging outside of the household during September-November; these three farms were all located in the Eastern region.

All of the Kosovar respondents reported feeding grain, while 44% reported feeding commercial feed. The commercial farm in Kosovo reported they fed grain and commercial feed, as well as hay and agricultural by-products. Hay was fed by 84% of respondents in Kosovo. Feeding butcher waste and food processing by-products was reported by 56.0% of respondents in Kosovo. Food scraps were fed by 80.0% of respondents from Kosovo; 100% of respondents reported the scraps were from their own household. One farm in Kosovo fed scraps from their own as well as another household. Additionally, one family farm reported feeding food scraps from a market. No farms reported boiling food scraps before feeding them to their pigs in Kosovo. All Kosovar producers kept pigs enclosed year-round, with no scavenging reported.

### Veterinary Care

North Macedonian respondents reported an average of 14.6 contacts (including phone calls) with their veterinarian per year. Commercial farms consulted with veterinarians (mean number consults: 26.9, SD: 26.6) approximately twice as often as backyard (mean number consults: 12.1, SD: 17.0) and family farms (mean number consults: 16.9, SD: 18.6). Eighty-five percent of farms reported they consulted a veterinarian when they had a sick pig, with 43.9% also separating sick pigs and 8.6% disinfecting pens. Only 4.2% of North Macedonian respondents reported treating animals themselves. No farms reported selling off sick pigs or their meat, though two North Macedonian family farms reported sending remaining healthy pigs to slaughter if others became ill. Four percent of farms in North Macedonia reported killing and disposing of sick pigs. Kosovar responses to sick pigs were similar, with 84% reporting they consulted their veterinarian and 56% separated sick from healthy pigs. Cleaning and disinfecting of sick pig pens was reported by 24% of respondents. In Kosovo, 68% of respondents reported treating sick pigs themselves. No sick pigs were reported to be slaughtered or sold in Kosovo.

When asked what they do when an adult pig dies, across North Macedonian farm types, the most common responses were disposal via burial (47.3%) or pit disposal (26.6%), followed by contacting their veterinarians (19.7%) or the veterinary authorities (12.7%). No respondents reported selling the meat of pigs found dead or feeding carcasses to other pigs. In North Macedonia, 2.7% farms reported feeding meat of pigs found dead to dogs In Kosovo, adult pigs that died were thrown away (88.0%), disposed of in a pit (28.0%), or buried (8.0%). The commercial facility in Kosovo reported they contact their veterinarians. No respondents reported selling the meat of pigs found dead or feeding carcasses to other pigs. In Kosovo, 20.0% of farms reported feeding meat of pigs found dead to dogs.

The most common vaccine used in North Macedonia is that for classical swine fever (CSF), 87.7% of farms reported administration. In North Macedonia, erysipelas is the next most common at 32.8%, with Aujezsky's disease and Pasteurellosis rarely reported at 2.6 and 1.1%, respectively. Approximately 10.5% of North Macedonian farms (all backyard and family farms) use no vaccines at all. In Kosovo, 96.0% of Kosovar producers reported using CSF vaccines; however, only the commercial facility reported use of any additional vaccines beyond CSF. One non-commercial Kosovar farm reported using no vaccines.

### Socioeconomics

In North Macedonia, the majority of farms reported pig rearing comprised only a proportion of the household income, with 29.1% of farms reporting all raised pigs were for home consumption only and only 11.6% of farms reporting pig rearing contributed more than 80.0% of the household income. Among backyard farms, 44.8% of pigs were reported to be raised for home consumption only, this number dropped to 2.7% for family farms. All of the producers interviewed in Kosovo reported household income from the pigs they raise (range: 2.0–80.0%). Removing the commercial farm, pig rearing contributed an average of 22.3% of household income on Kosovar farms.

About 19.5% of North Macedonian farms reported pig and/or piglet losses due to death on the farm or disappearance while free-ranging, with commercial farms having the highest proportion of respondents reporting such losses at 43.3%. In North Macedonia, results were similar for numbers of pigs reported lost to disease, with about 24.7% of farms reporting deaths due to disease. Approximately 66.7% of North Macedonian commercial farms report losses due to disease, vs. 18.5 and 28.1% of backyard and family respondents, respectively. Only 1.5% of respondents reported having pigs disappear or not return while they were free-ranging. These losses were reported by three backyard and four family farms, including two backyard farms that had advised their pigs were enclosed year-round. In Kosovo, 16% of respondents reported pig or piglet deaths on the farm (Kosovo has no free-ranging pigs and thus reported no deaths or losses while free-ranging); 88.0% of respondents reported pigs died due to disease.

### Pork Value Chain

**Figure 3:**
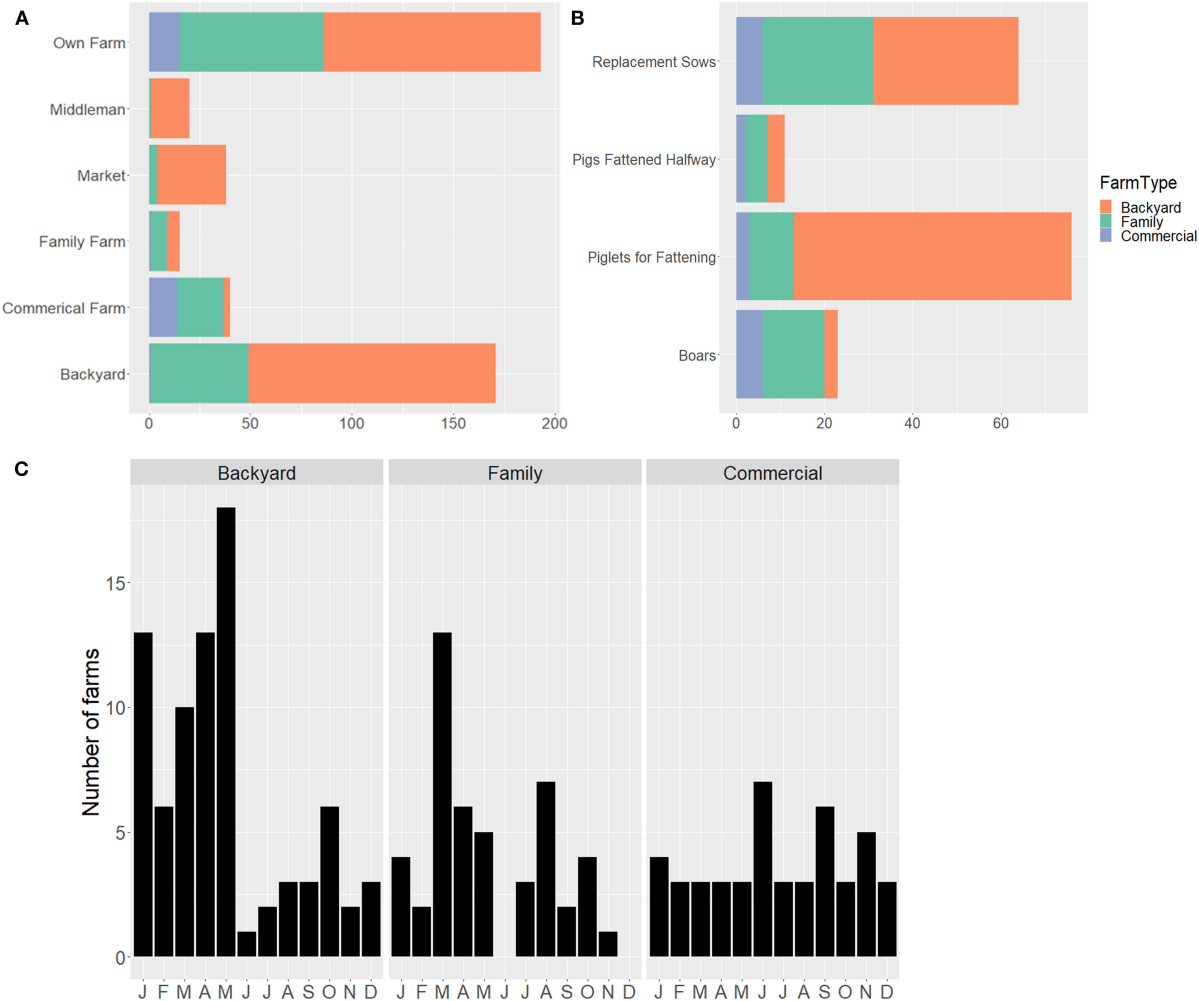
North Macedonian producer's pig buying practices by farm type by **(A)** source of pigs, **(B)** type of pig purchased, and **(C)** when pigs were purchased by month, based on questionnaires administered between September 2019-March 2020 (281 backyard, 146 family, and 30 commercial farms). Types of pigs: replacement sows = intact female pig for breeding; pigs fattened halfway = pigs over 25 kg but under market weight; piglets for fattening: pigs from weaning to about 25 kg; boar: intact male pig for breeding.

The majority of North Macedonian respondents reported buying or sourcing their pigs from backyard farms (37.4%) or their own farms (42.2%) (Figure 3A). The majority of commercial farms reported sourcing only from other commercial farms or their own facilities; however, in North Macedonia one commercial farm reported sourcing from backyard farms and one reported sourcing from a combination of family and commercial farms. In Kosovo, farms were more likely to source from non-commercial farms (64.0%), commercial farms (44.0%), and middlemen (28.0%), with only 12.0% sourcing from their own farms.

When buying in North Macedonia, the overall median number of pigs purchased was one. By farm type: backyard buyers bought a median of zero; family farms one; and commercial farms 21; with maximum purchases of 50, 200, and 25,000 for backyard, family, and commercial, respectively. Piglets for fattening (48.1%) and replacement sows (40.5%) were the most common types of pigs bought in North Macedonia (Figure 3B). Commercial farms buy throughout the year, while backyard and family farms tend to purchase early in the year (Figure 3C). In Kosovo, pigs for fattening (64.0%) and pigs fattened halfway (56.0%) were the predominate purchases, with replacement sows (28.0%) the next most common. Kosovar producers predominantly purchase their pigs at the beginning of the year: January (36%), February (52%), March (32%), April (12%).

**Figure 4:**
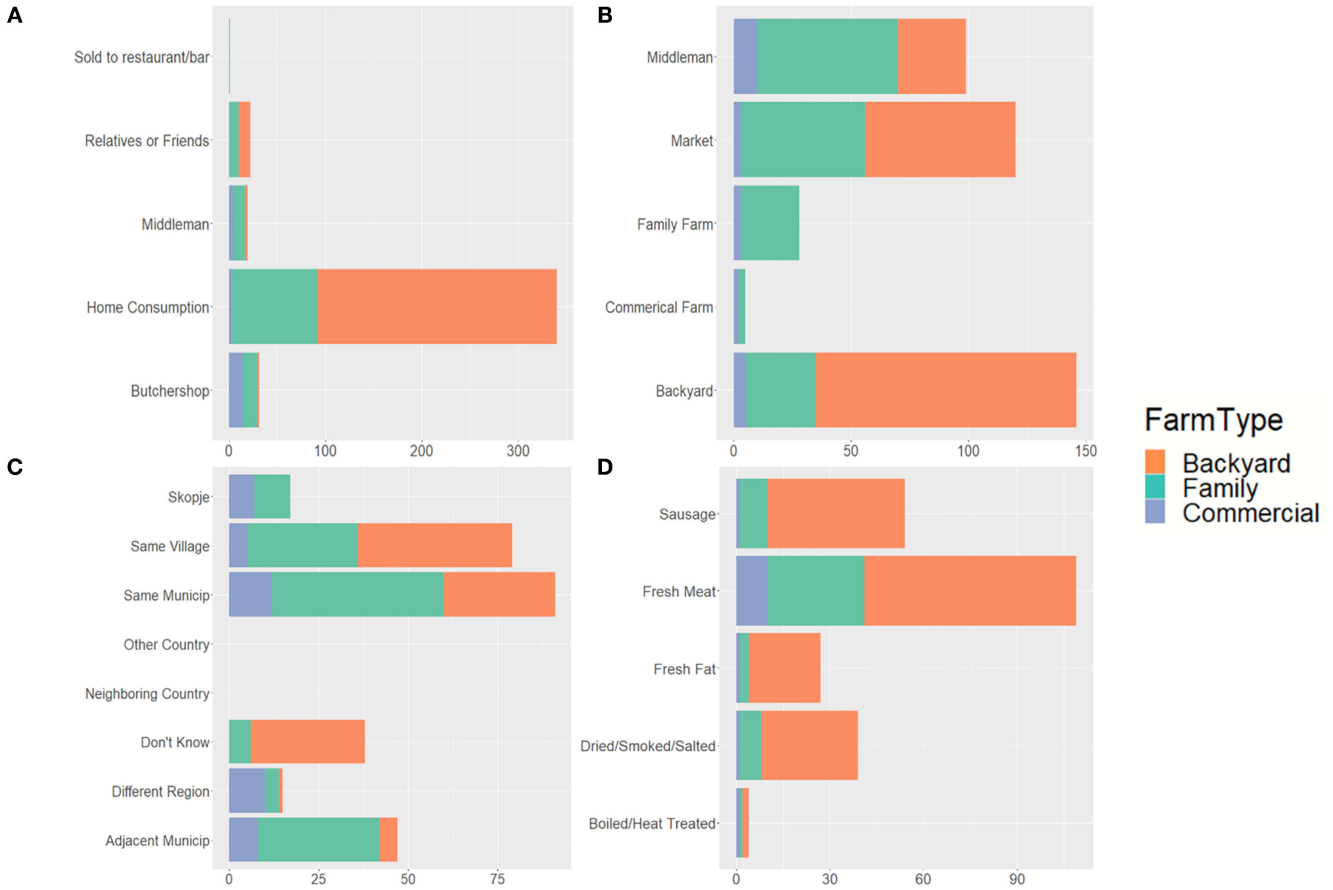
North Macedonian producer's pig selling practices by farm type by **(A)** fate of products sold, **(B)** who producers were selling to, **(C)** location of buyers, and **(D)** product type produced by farm, based on questionnaires administered between September 2019 and March 2020 (281 backyard, 146 family, and 30 commercial farms). One producer did report selling to pork products to Bulgaria. Due to survey wording, sausages cannot be differentiated as fresh vs. cooked or other.

The majority of backyard and family farms slaughtered their pigs at home, with 76.1% of North Macedonian farms reporting slaughter on-site by a family member (54.0%) or someone else (22.1%). North Macedonian farms slaughtering pigs at home overwhelming reported that they owned all the equipment used for slaughter or that the slaughterman brought everything needed. Only 2.1% of farms slaughtering pigs at home reported they borrowed all or only owned some equipment. Inedible materials from slaughter were primarily disposed of via offsite burial (33.6%) and pit disposal (26.1%) in North Macedonia. Sixteen percent of respondents in North Macedonia reported feeding inedible parts to dogs and cats. No respondents reported feeding parts to pigs. Fattened pigs were predominately slaughtered at the end of the year, with November the most common month across farm types, while the slaughtering of piglets had two peaks—April-May and November-January. Regarding the fate of pork products slaughtered at home, 90.2% of North Macedonian respondents reported the meat and products they produced were for home consumption, while most of the product from commercial farms ended up at butcher shops or with middlemen (Figure 4A). Backyard farms in North Macedonia reported they preserve (salt/smoke/dry) an average of 90.3% of meat slaughtered at home, with family farms reporting an average of 66.8%. This meat is then consumed over an average of 6.6 months for backyard farms and 4.5 months for family farms. Among those North Macedonian farms selling pigs, the majority reported selling to backyard farms (49.3%), markets (40.5%) and middlemen (33.4%) (Figure 4B). Almost all sales of meat and pork products were local. In North Macedonia this included sales within the same village (40.5%), same municipality (46.7%) or adjacent municipality (24.1%) (Figure 4C). One North Macedonian backyard farm located near the border reported sale of pork products in Bulgaria. In 19.5% of cases, North Macedonian sellers reported they were not aware of where their products ended up. In North Macedonia, fresh meat (87.9%) was the most common product sold or given away, followed by sausage (43.5%) and dried/smoked/salted meat (31.4%) (Figure 4D). Commercial farms sold consistently throughout the year, while backyard and family farms primarily sold at the end of the year (October-December).

About 64.8% of North Macedonian respondents answered questions regarding selling live pigs, suggesting there is a large segment of farms that do not sell pigs (this also corresponds with the numbers reporting production for home consumption only). The pigs sold in North Macedonia were primarily ready-to-slaughter pigs (50.9%) and piglets for fattening (69.4%). In a given year, North Macedonian commercial sites reported selling a median of 1,128 pigs (mean: 4,570, SD: 7,561, range: 0–24,000), compared to backyard and family farms with medians of 1.0 (mean: 7.0, SD: 14.1, range: 0–80) and 27.5 (mean: 150.0, SD: 587, range: 0–6,404) pigs sold, respectively.

All responses from Kosovo reported slaughter on-site, with approximately half of slaughter performed by family (47.8%) and half by someone else (52.2%). Having all the equipment needed for slaughter was reported by 39.1% of respondents, while 47.8% borrowed or shared with neighbors. In Kosovo 95.7% of respondents reported inedible materials from slaughter were fed to dogs and cats, 43.5% disposed of via pit disposal and/or 39.1% thrown offsite. The commercial farm in Kosovo reported off-site burial or collection. No respondents reported feeding parts to pigs. Fattened pigs were reportedly slaughtered in October (25%), November (100%) and December (50%). Piglets were slaughtered in May (18.2%), June (77.3%) and July (45.5%). Pork products from homeslaughter were predominantly for home consumption (100.0%), or sold or given to relatives, friends and family (80.0%); however, pork products were also reportedly sold to middlemen (32.0%) and restaurants or bars (16.0%). Sale of pork products was primarily local, sold in the same village (100.0%), same municipality (95.4%), or adjacent municipality (52.4.0%). Pork products were also sold Skopje (28.6%). Fresh meat (100.0%), dried/smoked/salted meat (81.1%), fresh fat (38.1%), and sausage (14.3%) were the most commonly sold or gifted pork products.

About 44.0% of Kosovar respondents answered questions regarding selling live pigs. Among those selling pigs, 81.3% reported selling to backyard farms, followed by middlemen (54.5%) and family farms (27.3%). No respondents reported selling pigs to commercial farms or markets. The majority of pigs sold in Kosovo were ready-to-slaughter pigs (63.6%), piglets for fattening (54.5%) and pigs fattened halfway (45.5%). Pigs were primarily sold during October-November and April-June.

### Biosecurity

#### Basic Biosecurity

Producers were asked about a variety of biosecurity and sanitation practices on their farms. Over 90.6% of North Macedonian producers reported that their home or farm was fenced, with 98.2% reporting that their pig pens were fenced. Only 23.4% of North Macedonian producers reported isolating newly purchased pigs; of those who do isolate, the mean time was 24.9 days (SD: 12.2, range: 1–60). Even among commercial farms, the isolation of new pigs was not reported to be consistently practiced (46.7%). Equipment lending or borrowing between neighbors was reported by only 3.7% of respondents in North Macedonia, with commercial farms never lending or borrowing equipment. Changing shoes (94.1%) or clothes (92.8%) before going to the pigs was common in North Macedonia, with hand washing before going to the pigs being slightly less consistent (87.1%). Disinfection mats were used less reliably (68.5%). In general, commercial farms were the most consistent with their biosecurity practices, with all farms reporting fenced properties, fenced pig pens, and consistent practices of changing shoes and cloths, washing hands and using disinfection mats before going to pigs.

In Kosovo, 100% of respondents reported their farm/home was fenced; 92% reported their pigs were kept in a pen or fenced in. Among Kosovo respondents 40.0% reported isolating new pigs. Sharing of equipment was reported by 72.0% of respondents. In Kosovo, changing clothes (40.0%) and washing hands (28.0%) were performed less frequently than in North Macedonia; only the commercial farm used disinfection mats.

### Visitors to Farm

Next, producers were asked about the exposure of their pigs to people visiting the farm and pigs from other premises. Veterinarians were the most common persons allowed access to pigs at 86.7% in North Macedonia. Twenty-three percent of North Macedonian farms had restricted access, with no one allowed near the pigs. Friends (9.0%), neighbors (8.5%), and buyers (8.1%) were each allowed in at a low rate. Slaughtermen had access at 4.2% of farms in North Macedonia. Only 1.8% of North Macedonian farms allowed fellow pig farmers access to their pigs. Commercial farms were generally the most restrictive, with 36.7% allowing no access and 56.7% only allowing access to veterinarians; one North Macedonian commercial farm reported allowing fellow pig farmers and one allowed buyers onsite. In Kosovo, veterinarians were allowed on 100.0% of farms. Among Kosovar respondents 28.0% allowed neighbors, 36.0% allowed buyers, and 28.0% allowed slaughtermen, to access their pigs. Fellow pig farmers were allowed access by 76.0% of Kosovar respondents.

### Pigs From Outside the Premises

Bringing in external boar to cross with sows was reported by 8.6% of respondents in North Macedonia, including three commercial facilities. Most North Macedonian farms reported either using artificial insemination (35.9%) or owning their own boars (35.9%). Only 2.9% of farms, and only backyard and family farms, reported taking their sows offsite for breeding. Of the Kosovar farms assessed, 40.0% did not have breeding animals on-site; among those who did, 32.0% brought in an external boar, 12.0% sent their sows offsite, and 12.0% had their own boar. Artificial insemination was only reported by the commercial farm in Kosovo.

In North Macedonia, only 3.9% of farms reported having seen wild boar in the proximity of the farm in the last 12 months, with most sightings occurring late in the year. Wild boars were reported throughout the year in the Northeastern region, in November in the Eastern region, and in October and December in Vardar. Those farms who had seen wild boar were all in the eastern regions of the country. Among pig producers, 2.4% in North Macedonia reported hunting wild boar. Only one farm in Kosovo reported seeing wild boar. Hunting wild boar was reported by 8.0% of Kosovar respondents.

### Waste Disposal

Most farms in North Macedonia reported their household waste was collected by the municipality (77.2%). In North Macedonia, burning (9.2%) and throwing/dumping household waste off-site (8.1%) were the next most common disposal routes, with on-site burial of waste rarely reported (3.1%). All but one commercial farm report waste removal by the municipality. No farms reported burying off-site or discarding household waste on their premises. One third of North Macedonian farms reported that there was no disposal site available for household waste in their village. In North Macedonia, most village disposal sites were fenced sites (46.8%), with unfenced sites less common (11.1%). Burial (2.5%) or burning (5.8%) of household waste at village disposal sites was rare. In Kosovo, 68.0% of respondents reported household waste was collected by the municipality, with discarding household waste offsite the next most common form of disposal (36.0%). One farm reported burning some of their household waste. No disposal site available for household waste in the village was reported by 80.0% of Kosovar respondents; 12.0% reported a fenced disposal site, 4.0% a non-fenced disposal site, and 4.0% burial at the disposal site. No burning of waste at village disposal sites was reported.

Manure was most commonly disposed of in unfenced (49.2%) or fenced (27.8%) gardens or fields, or stored on-site (36.5%) in North Macedonia. Rarely manure was disposed of at a dumpsite (8.3%). It was very uncommon to sell or give away pig manure (1.3%) in North Macedonia. In Kosovo, manure disposal was highly variable: 84.0% dump off-site, 36.0% spread in unfenced fields, 32.0% sell or give away, 20.0% store in a pit, and 8.0% spread in fenced fields.

### Advanced Biosecurity (Only North Macedonian Family and Commercial Farms)

A second series of biosecurity questions was targeted at family and commercial farm operations: 39.2% of farms reported having a double fence; 55.5% reported having separate clean and dirty areas for employees; and 42.1% reported restricting the kind of food products employees could bring on-site for their own consumption. No commercial farms allowed workers to keep their own pigs at home, with 86.8% of all respondents reporting workers could not keep pigs. Similarly, all but one commercial farm reported their workers were not allowed to hunt in their free time, with 91.1% of all respondents not allowing workers to hunt.

When asked about having detailed disinfection protocols, 55.3% reported protocols for vehicles, 68.8% for equipment, and 65.2% for people. Eighty-nine percent of commercial farms reported protocols in place for vehicles, equipment and people, compared to 41.2% of family farms.

About one third of farms report never re-assessing their biosecurity procedures. However, 27.1% were reassessing each month, with 18.6% doing so every 3 months, and 10.9% twice a year. Commercial farms were more likely to reassess more often.

Forty-three percent of farms reported never organizing events to educate workers about ASF; however, 14.6% did so each month, 12.3% every 3 months, 15.4% every 6 months, and 14.6% once a year. Commercial farms organized training more often.

### ASF Awareness

Producers were asked a series of questions regarding where they get information on animal diseases, their level of concern, and to test their knowledge of ASF. The most common sources of animal health information in North Macedonia were veterinarians (96.3%), television (75.6%), the internet (39.4%), and leaflets (29.8%). No one reported getting animal health information at church. These responses were consistent with responses about where producers heard about ASF. One percent of North Macedonian producers report not having heard of ASF—this represents three backyard farms, three family farms and one commercial farm. Reported sources of animal information were similar in Kosovo: veterinarians (96.0%), television (72.0%), local authorities (48.0%), newspapers (32.0%), leaflets/posters (20.0%). Among the Kosovo respondents, 32.0% reported not having heard of ASF.

Given a list of pig diseases—ASF, Aujezsky's disease, classical swine fever (CSF), foot-and-mouth disease (FMD), porcine reproductive and respiratory syndrome virus (PRRS), swine influenza, Seneca Valley virus (as a control; has not been reported in the region)—producers were asked to rank the top three diseases of most concern. African swine fever (85.6%), CSF (85.3%) and swine influenza (41.4%) were the predominant diseases of concern in North Macedonia. While ASF and CSF were consistently of concern, the remaining diseases showed some regional variation. In Kosovo, 68.0% of farms did not list ASF in their top three disease of concern, rather CSF (92.0%), swine influenza (92.0%), and FMD (68.0%) predominated.

In recognizing the signs of ASF, the most commonly reported signs from North Macedonian producers were: hemorrhages on the skin (60.6%), reduced appetite (60.0%), fever (60.0%) and sudden death (52.1%). Only 2.4% reported not knowing the signs of ASF, consistent with the previous numbers who had reported not hearing of ASF. Only 1.5% of producers thought ASF was zoonotic. The most common North Macedonian responses regarding the ways their pigs might contract ASF were: introduction or exposure to diseased animals (87.1%), fomites, e.g., infected boots or cloths (49.9%), and feeding infected pork products (39.2%). Twenty-four percent were concerned about transmission routes not relevant to ASF, such as 20.4% mosquitoes, 3.5% wind and 1.8% bad vaccines. In Kosovo, the most commonly reported clinical signs related to ASF were fever (68.0%), diarrhea (64.0%), reduced eating (44.0%), and sudden death (40.0%). Kosovar respondents reported diseased animals (76.0%), feeding infected pork products (28.0%), and fomites (20.0%) as paths of ASF transmission. Twenty percent of respondents did not know how ASF could infect their pigs.

When it comes to reporting suspect ASF cases, 76.4% of producers in North Macedonia reported they would quickly report ASF to veterinary authorities if they suspected it on their farms. Twenty-three percent in North Macedonia advised they would wait a few days to report due to concerns about it being a false report. In North Macedonia, only two farms would wait a few days to report to the veterinary authorities due to concern for financial losses. In Kosovo, 48.0% of respondents said they would quickly report suspect ASF, 12.0% would wait a few days due to concerns about a false report, and 40.0% would wait due to concern for financial losses.

Finally, when asked why an owner may not report ASF, producers in North Macedonia reported not knowing how to report (39.6%), being unclear about what might happen after reporting (31.1%), the culling of their pigs (27.8%), the subsequent restriction of sale of their pigs (24.3%), damaged reputation (15.1%), and no compensation (9.8%), as the top reasons. Only 2.4% said the owner would prefer to deal with the disease themselves. Reporting being too time consuming was only cited by 0.8% of respondents. In Kosovo, 60.0% reported not knowing how to report, 64.0% were concerned about post-reporting unknowns, 36.0% were concerned about banned sales, 28.0% felt reporting was too time consuming, 20.0% were concerned about their reputations and 16.0% were concerned about their pigs being culled.

### Biosecurity Risk Scores and High-Risk Areas for ASF Introduction

**Figure 5:**
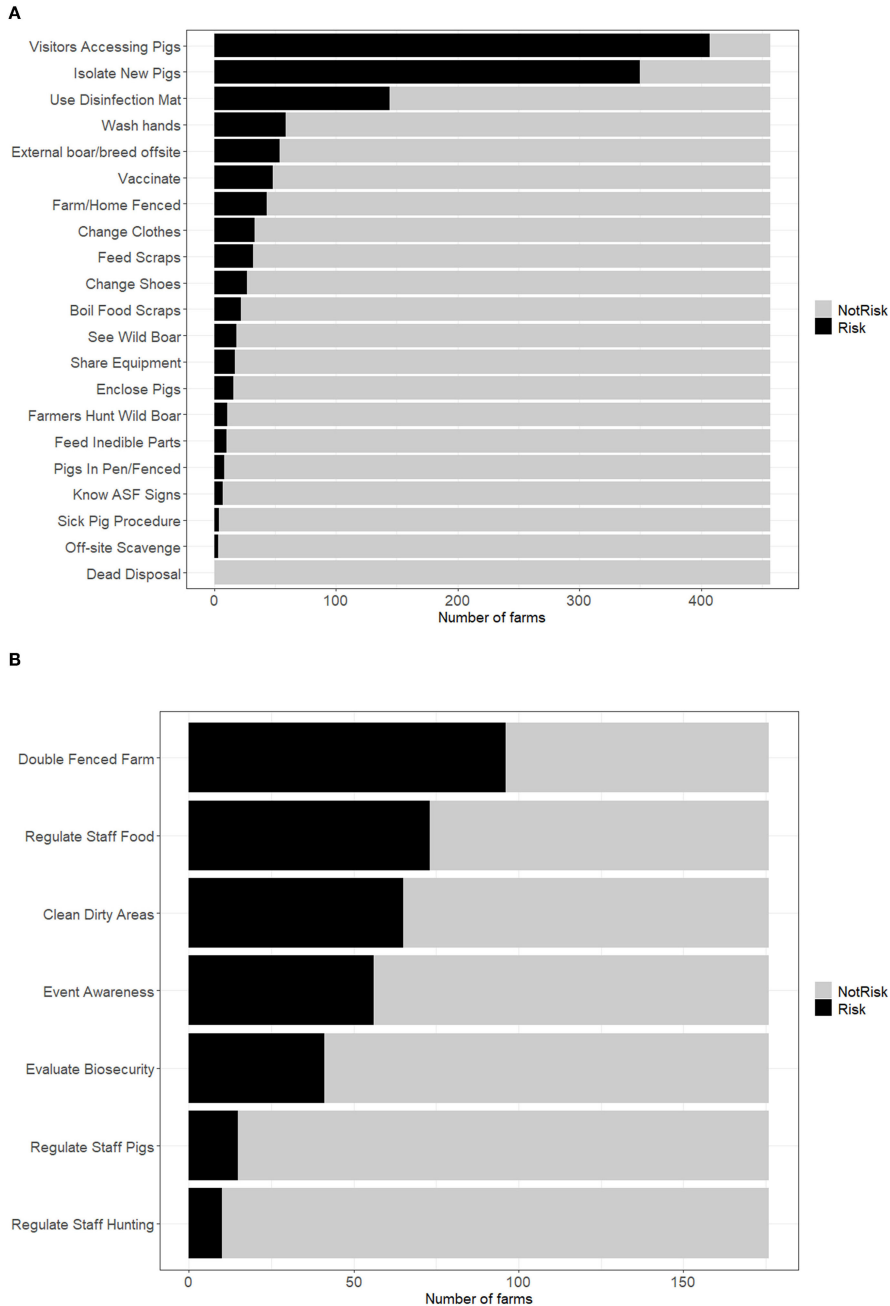
Dichotomous scoring responses for questions administered to North Macedonian pig producers between September 2019 and March 2020, characterizing reported practices as no/low risk vs. contributing risk for ASF introduction based on biosecurity characteristics for **(A)** all farms, and **(B)** family and commercial farms. Scores were used to calculate biosecurity risk scores. “Not risk” answers were assigned a score of zero, “risk” answers were assigned a score of one. Two separate sets of biosecurity risk scores were developed to account for additional information provided in a subset of biosecurity questions that was only answered by family and commercial farms.

A subset of survey questions was selected to reflect the biosecurity practices and associated risk level of each farm in North Macedonia. The responses to these questions were dichotomized into low/no risk or contributing risk based on whether a farm performs or does not perform certain activities, e.g., vaccinating vs. not vaccinating pigs (Supplementary Table 1). The distribution of these answers is presented in Figure 5. The most common high-risk practices reported were allowing visitors (e.g., veterinarians, fellow pig farms, buyers, neighbors, friends) to access the farm, failure to isolate new pigs, and not using a disinfection mat. Among those questions targeted to family and commercial farms, more variability in answers was noted, with the most common high-risk practices including: not having a double fence, not regulating the food workers bring on the farm, not having separate clean and dirty areas, and not having events in which to educate and increase the awareness of employees about ASF.

**Figure 6:**
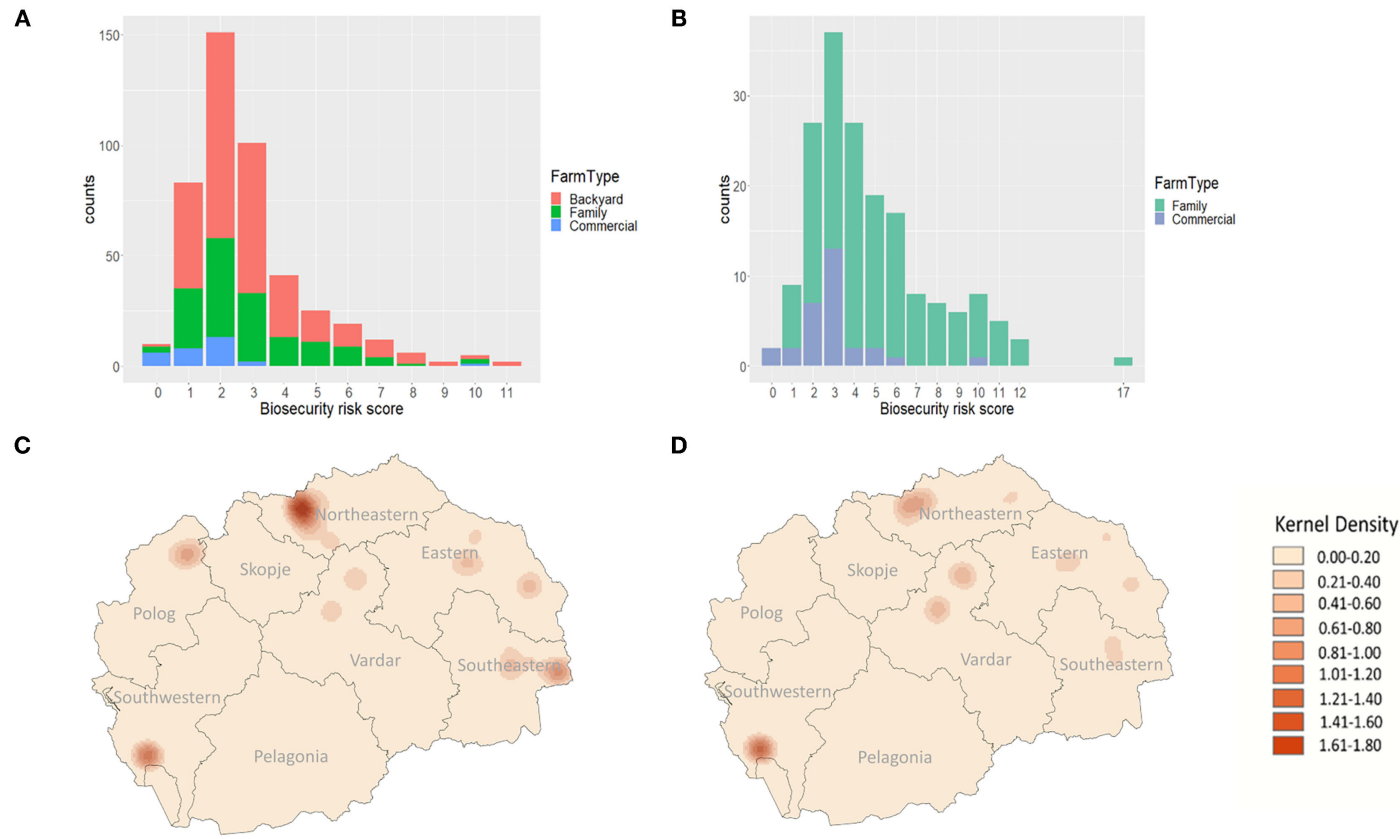
Biosecurity risk scores for **(A)** all pig farms, and **(B)** family and commercial pig farms, administered questionnaires in North Macedonia between September 2019 and March 2020. Biosecurity risk scores represent a non-weighted linear combination of values assigned to dichotomized survey questions in which higher scores representing higher risk. Kernel density estimation (KDE) mapping of biosecurity risk scores for **(C)** all pig farms, and **(D)** family and commercial pig farms administered questionnaires in North Macedonia between September 2019 and March 2020.

Most farms have low biosecurity risk scores—indicating low risk of disease introduction and good biosecurity (Figure 6). When evaluating scores across all farm types, the highest biosecurity risk scores (those with the worst biosecurity) were generally observed among backyard and family farms. In both the all-farm and family and commercial focused assessments, commercial farms tended to score better (lower) than other types of farms (Figure 6).

**Figure 7:**
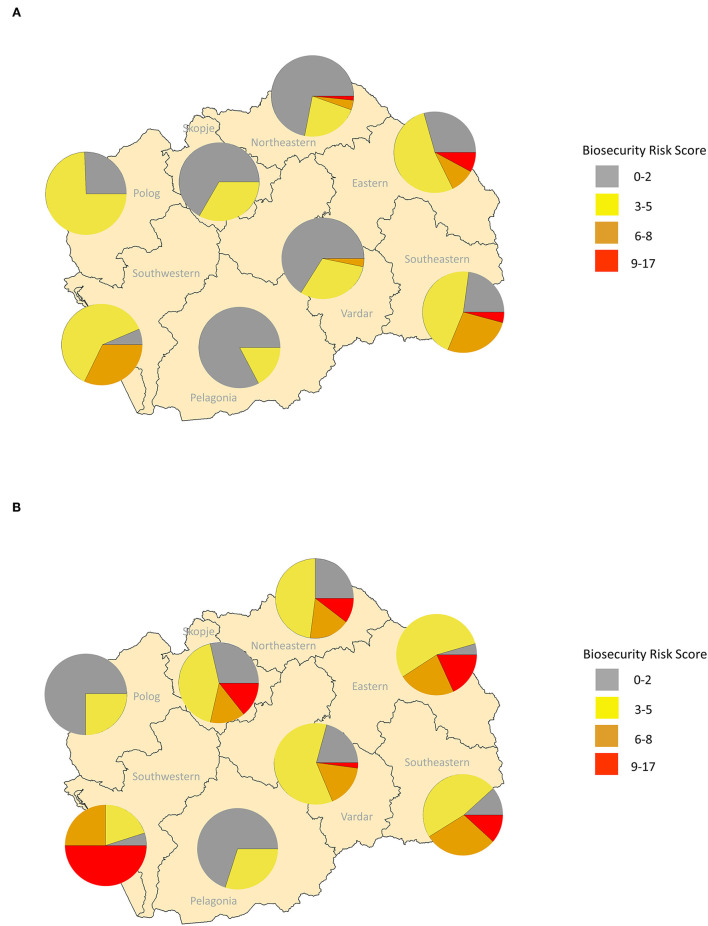
Mapping of biosecurity risk score by North Macedonian region. Pie charts represent the proportion of pig farms with the corresponding biosecurity risk scores in each region for **(A)** all farms, and **(B)** family and commercial farms. Biosecurity risk scores represent a non-weighted linear combination of values assigned to dichotomized survey questions collected between September 2019 and March 2020 in which higher scores represent higher risk.

Risk maps generated using the all-farm biosecurity risk scores, identified areas of high risk for ASF introduction in the Northeastern, Southwestern, and Southeastern regions of North Macedonia (Figure 6C). When focusing on family and commercial farms, the Southeast region's focus is no longer highlighted and the Eastern region becomes lower in risk (Figure 6D); however, the high-risk areas in Northeastern and Southwestern regions remain. While the KDE maps identified high risk areas in the Northeastern and Southwestern regions, those individual farms with the highest biosecurity risk scores were located in the East, with the Southeastern region having the largest proportion of high-risk scoring farms (Figure 7A). Among the family and commercial farms subset, the highest individual scores were observed in the Northeastern and Eastern regions, with a high level of variability observed in the Southwestern region (Figure 7B). Among this subset, the Eastern and Southwestern regions have the highest proportions of high-risk biosecurity risk score farms.

### Generation of Farm Profiles Based on MCA and HCPC

**Figure 8:**
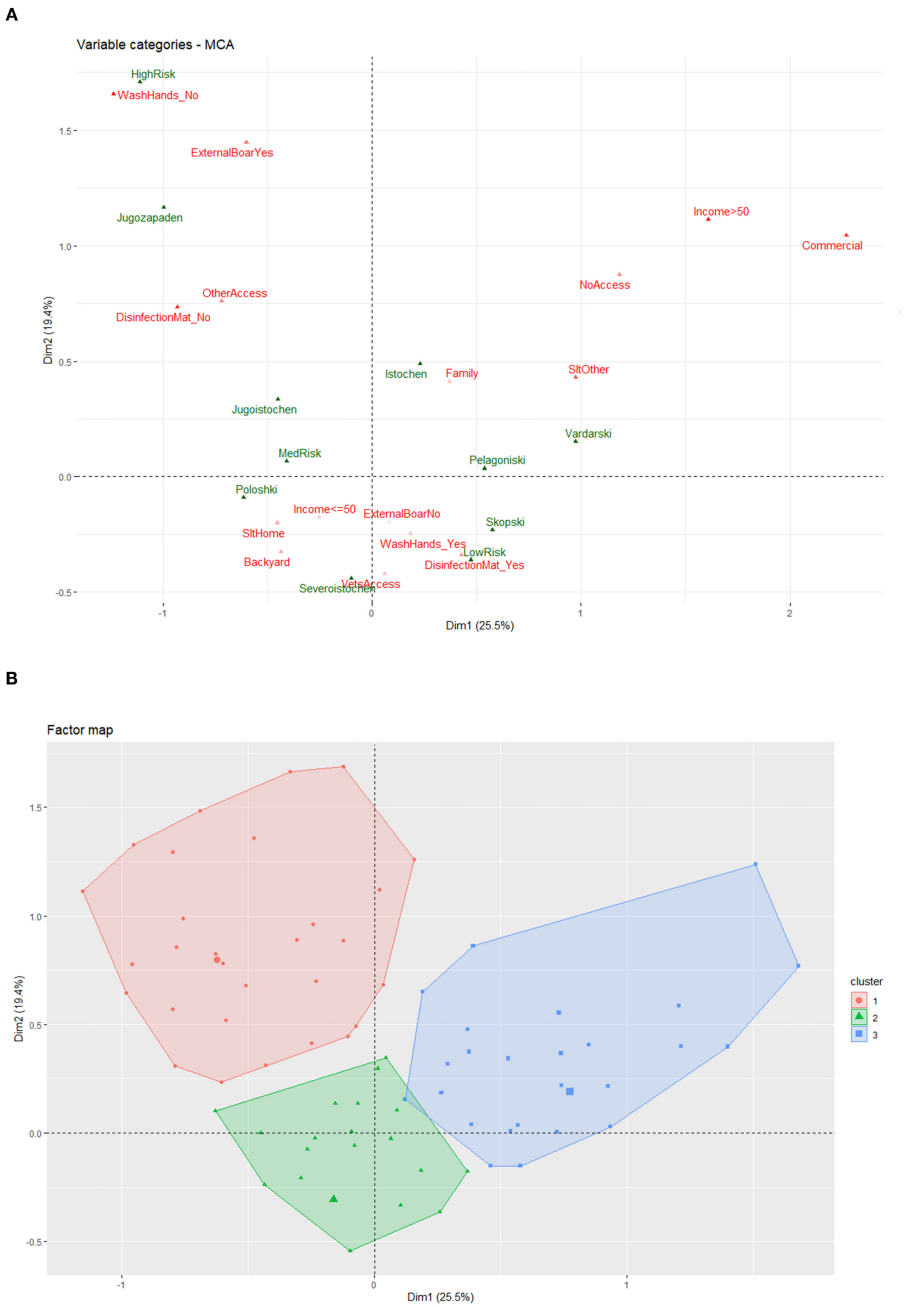
Multiple correspondence analysis (MCA) and hierarchical clusters of principal components (HCPC) results for North Macedonian pig farms using farm characteristics and practices reported in questionnaires administered between September 2019 and March 2020, with region and biosecurity risk score categories used as supplemental variables. **(A)** Graph of the correlation of categorical variables by dimension. The distance between points gives a measure of their similarity; variables that group together have similar profiles. The distance from the axis represents the level of correlation that variable has with the given dimension; variables near the origin have low correlation with either dimension. Red: analyzed variables, Green: supplemental variables. Variables: WashHands_Yes/No: wash hands before going to pigs, External Boar_Yes/No: allow interaction with external pigs, DisinfectionMat_Yes/No: use disinfection mat, SltHome/SltOther: slaughtered for home consumption vs. other, NoAccess/VetAccess/OtherAccess: allow no access to pigs, allow only veterinarians to access pigs, allow other people (neighbors, buyers, fellow pig farmers) to access pig, Income ≤ 50/Income > 50: household income from pig rearing ≤ 50 vs. >50%, Commercial/Family/Backyard: farm type. **(B)** Plot of HCPC results. HCPC groups respondents into clusters based on their similar response profiles. Our analysis generated three clusters. The red cluster corresponds to high biosecurity risk farms, and groups respondents who reported not washing hands before going to pigs, allowing external pigs on the farm, allowing visitors other than veterinarians to access pigs and not using disinfection mats. The blue cluster groups respondents with profiles including commercial farms, household income from pigs >50%, not allowing visitors to access pigs, and slaughter done by someone outside the household. The green cluster groups the remaining respondents whose responses were not highly correlated with either dimension.

MCA grouped not washing hands, allowing access to external boar, allowing access to people other than veterinarians, and not using a disinfection mat as variables highly correlated with dimension 2 and high biosecurity risk scores (Figure 8A). Low and medium biosecurity risk scores were more difficult to delineate, as factors grouped around the X-Y axis did not strongly contribute to differentiating farms for these dimensions. Commercial farms grouped with pig rearing being more than 50% of household income, allowing no people to access pigs, and slaughtering for a purpose other than home consumption as variables highly correlated with dimension 1. Hierarchical clustering identified three separate groups of respondents with similar profiles, or pattens of responses to questions about their farm practices (Figure 8B).

## Discussion

This study provides the most complete profile of the pig industry in North Macedonia available, covering 77.7% of the pig population in the country, thanks to the large sample size and the comprehensive survey responses from pig producers on their husbandry practices, the pork value chain, biosecurity practices, and disease awareness. The recent ASF introductions into Bulgaria, Greece, and Serbia, highlight the need to better understand the pig sector in this region and to inform future targeted interventions. Like other countries in the Balkans, North Macedonia and Kosovo have numerous risk factors for ASF introduction including many low biosecurity small holder farms, free ranging pigs, farms practicing swill feeding, high wild boar suitability, and high connectivity to ASF positive countries through international travel (33, 34). This study has provided an in-depth description of the North Macedonian pig sector, contrasted these practices with those in Kosovo, and highlighted target areas for disease risk mitigation efforts.

North Macedonian farms had a high rate of turnover among their pigs; this is consistent with census data that shows a relatively large proportion of small farms do not maintain pigs year-to-year, making registration of, and outreach to, these small holder farms a challenge. The predominant use of commercial feed (97.2%) and grain (38.7%) suggests sites selling pig feed may provide good venues to access producers. The reports of feeding scraps and inedible parts to dogs and cats poses a zoonotic concern, not for ASF, but for other diseases such as pseudorabies or echinococcosis. Education on the risks of feeding food scraps to pets, and their role in the transmission of zoonoses, could be added to materials targeting swill feeding.

The North Macedonian pig sector seems to make good use of their veterinarians and to trust them as an information source (96.3%). However, only a third of producers called their veterinarians or the veterinary authority when they had pigs die. This should be highlighted as a major gap in current passive surveillance, a critical element for early detection and eradication. Burial and pit disposal predominated as methods of dead pig disposal; depending on the depth of burial, these methods should limit the access of wildlife to carcasses. The last outbreak of CSF in North Macedonia occurred in 2008 (1), yet vaccine compliance remains high. The vaccination campaign, financially sponsored by the state for farms with fewer than ten pigs, was suspended in October 2019 and North Macedonia is currently in the process of applying for CSF-free status. No other vaccines are compulsory. This history of vaccine compliance suggests that if an ASF vaccine were to become available, North Macedonia could expect high compliance from its producers, especially if financially backed. However, it should be mentioned, the initial phases after discontinuing a vaccine campaign are challenging, in that cases of ASF may be mis-diagnosed as re-emerging CSF. Diagnostic confirmation will be especially critical in differentiating the cause of illnesses among cases with similar clinical presentations.

A large number of households report raising pigs for home consumption and as a source of supplemental income. This reliance on pigs to feed families, as well as contribute to household income, highlights the extent to which an ASF introduction would impact the food and financial security of these producers. Adequate indemnity programs and education about these programs will be needed to support producers and get buy-in on timely disease reporting. Commercial farms reported higher rates of death and disease than backyard and family producers. These systems should be evaluated for potential husbandry, health (e.g., vaccination) and biosecurity interventions that may reduce these losses.

The pork value chain is predominantly localized, which may limit disease spread if ASF is introduced (35). The sale and slaughter of pigs is also highly seasonal. Religion and cultural habits may influence these patterns as well as the probability of ASF introduction into domestic pigs. Serbians in the North, and Catholic Albanians in the West, keep pigs and may have different practices and seasonality in their pig rearing and trade. The large concentration of Muslims in Western North Macedonia likely contribute to the low density of pig farms in this area.

Biosecurity is highest among commercial farms, but sanitary practices were in general fair to good. The primary areas that could consistently be improved upon would be the use of disinfection mats, the creation of separate clean and dirty areas, and the implementation of consistent disinfection protocols. The efficacy of disinfection mats and boot baths is dependent on removal of visible debris before use, and the use of appropriate disinfectants at adequate concentrations and for enough time (36, 37). While effective when used properly (36, 38), successful implementation of disinfection mats in small-holder settings may be a challenge due to lack of funds for disinfectants, rapid soiling, and improper protocols. Isolation of new pigs was reportedly uncommon—this may be associated with a lack of space, all-in all-out practices, or low perceived value. However, the overall percentage of producers reporting separating sick pigs was higher than that reporting isolating new pigs—suggesting that while areas for complete isolation may not exist, some level of separation may be possible. In general, most farms did not allow visitors near their pigs. Backyard and family farms were most likely to allow visitors to their premises to access their pigs. Training and future outreach should continue to highlight the risk of new pigs and visitors introducing disease. Visitors accessing pigs/farms was identified as a significant risk factor for disease introduction to backyard farms in Romania, and a case study of a backyard farm in Bulgaria cited visitors as the most likely route of ASF introduction (39, 40). Enclosure of pigs, and the removal and treatment of trash by the municipality, should help restrict wild-domestic pig interfaces contributing to disease exposure. While very few wild boar sightings were reported, ASF introduction via wild boar was listed as the highest risk pathway for Eastern Europe by recent studies (41). Outbreaks in wild boar in Bulgaria and Serbia confirm this risk in the region. Additional data on wild boar populations in these countries is needed.

Addressing hurdles to timely reporting is critical to a country's disease detection. Kosovar producers reported a high level of concern about the financial implications of reporting, suggesting the need for clear messaging and planning around indemnity for animals culled to control disease. In both North Macedonia (39.6%) and Kosovo (60%), producers reported not knowing how to report suspect ASF, while about a third of respondents in each country were concerned about post-reporting unknowns, culling, and restricted sale of pigs. Concern about reputation or attempting to control disease oneself, was less commonly reported than previous studies in the region have shown (42). These results indicate the need for transparency and communication about reporting. North Macedonia is in the process of improving their national surveillance programs. While they have ASF and CSF programs designed, they have not been widely implemented. The country currently relies heavily on passive surveillance, and the use of government authority to place quarantines during disease investigations. This heavy reliance on passive surveillance further emphasizes the need for education about diseases of concern, how to prevent disease introductions (e.g., biosecurity), what to look for, how to report, and what to expect during a disease investigation.

Our biosecurity risk scores and KDE maps highlight specific areas for targeted intervention. On the KDE maps we observe diminishment of the foci in the Southeast and Eastern regions, while retaining the foci in the North and West, when focusing on family and commercial farms vs. focusing on all farms, indicating that high biosecurity risk scores from family and commercial farms were contributing to high risk of ASF introduction in the North and Southwest, while backyard farms likely have a more important role for risk in the South and East. While the highest biosecurity risk scores were focused in the East, Southeast and West, our KDE maps register the highest risk areas in the West and North. This may be due to the small number of farms with high biosecurity risk scores and KDE being influenced by the number of farms in an area, particularly in the North; future work could consider standardizing biosecurity risk in a region by the number of farms in that region. Outreach for backyard farms at high risk of ASF introduction should be targeted in the East, particularly in Southeastern region. More general campaigns to reach all farm types are warranted in Southwestern, Northeastern and Eastern regions. Primary areas in which improvements could be made include: isolating/separating new pigs, using disinfection mats, and limiting access of visitors to pigs. Among family and commercial farms, investment in double fencing, separate clean and dirty areas, and educational training would improve current biosecurity risk scores.

MCA and HCPC divided farms into three groups—dimension 1 which captured commercial farms, dimension 2 which captured farms with high-risk practices, and a third group made up of the remaining farms. Our analysis suggests that farms with certain high-risk behaviors were likely to have profiles that demonstrated multiple risky behaviors resulting in an overall high biosecurity risk score profile. The specific behaviors that were highly correlated with dimension 2—not washing hands, allowing visitors including friends, neighbors, buyers, and slaughtermen, and external pigs onto the farm, and not using a disinfection mat—were correlated with high-risk biosecurity risk scores. This grouping generated a profile of responses to this subset of questions. Farms with similar responses are expected to have poor biosecurity practices, and thus high biosecurity risk scores, and should be targeted for education and improved biosecurity, i.e., a farm that does not practice regular handwashing before working with their pigs likely has other poor biosecurity habits, will likely have a high biosecurity risk score, and should be targeted for intervention.

The Kosovo pilot study was intended to gain awareness of practices in their pig sector to support the expansion of FAO activities, including biosecurity training that is actively under development. The low sample size from the pilot study in Kosovo implies we should interpret these results with caution. However, a few marked contrasts between North Macedonia and Kosovo, that may impact the risk of ASF spread, should be noted. Kosovo has good, consistent practices around keeping pigs confined and not allowing scavenging. However, Kosovar pig producers reported a much higher rate of swill feeding, and not treating food scraps that were fed to pigs. These responses indicate that while swill feeding is banned in surrounding European Union countries, it is still widely practiced in this region and should be highlighted as a topic for education campaigns (33). In general, losing pigs to illness was more widely reported in Kosovo than North Macedonia. The disposal of inedibles from slaughter and dead pig carcasses as thrown offsite and fed to dogs, could provide access from wildlife. More visitors and pigs from other farms were allowed on-site, and manure was moved offsite through sale and disposal methods, providing the means for disease introduction and spread. One third of respondents said they had not heard of ASF (compared to 1.5% in North Macedonia), and it was not reported as a top disease of concern from Kosovar producers. All of this suggests that education campaigns targeted at informing producers about ASF, its introduction pathways, clinical presentation, and how to report and seek aid, could improve early detection and reduce disease dissemination risk among these producers. The best means of reaching pig producers is through their veterinarians and television; North Macedonians also used the internet, while Kosovars preferred newspapers.

With data collected via a questionnaire, this study is subject to reporting bias by the respondents. In North Macedonia in particular, with questionnaires being administered by veterinarians, producers may have been more likely to report higher usage of veterinarians, higher levels of care, and stricter biosecurity practices. Additionally, outreach and educational campaigns targeting ASF awareness have been ongoing since 2018, which may have led producers to change or at least report higher quality practices. FAO training did occur in September, October, and November of 2019, while the initial phases of the survey were underway; however, these trainings were primarily targeted at veterinarians vs. producers and are not thought to have had much impact on the respondents. Survey responses are being used to inform updates and development of training materials for producers in the region. In the calculation of the biosecurity risk scores, non-answers were assigned a value of zero. This practice may have resulted in an underestimation of the biosecurity risk scores for some farms.

Overall, this study has provided a thorough review of the practices of the pig sector in North Macedonia, highlighting some similarities and contrasts with neighboring Kosovo, and discussing the potential strengths and vulnerabilities regarding the risk of ASF introduction and spread. We have highlighted some specific aspects (and regions) for improvement via additional and targeted educational campaigns and risk reduction interventions. This information will be of great value to inform risk assessments of ASF introduction/exposure, and modeling of ASF spread, if it is eventually introduced into the country. Ultimately, all of these tools will contribute to better prevention, early detection, and control efforts for ASF in North Macedonia and Kosovo.

## Data Availability Statement

The raw data supporting the conclusions of this article will be made available upon request.

## Author Contributions

KO'H performed the initial draft preparation, data curation and validation, development of the R-code, and formal analysis under supervision of BM-L. DB-A, MH, and BT developed the questionnaire and initiated recruitment of farms included in the study, collected and organized the raw data, and contributed to data cleaning and validation. BM-L supervised the development, implementation and interpretation of the analytic approach. All authors contributed to the project conceptualization and critical and extensive review and editing of the submitted manuscript.

## Funding

Data collection was conducted by FAO through the Technical Cooperation Programme (TCP) (TCP/RER/3704 and TCP/KOS/3703). Data analysis was supported by The Ecology and Evolution of Infectious Diseases Program, Grant No. 2019-67015-28981 from the USDA National Institute of Food and Agriculture and the UC Davis Graduate Group in Epidemiology.

## Author Disclaimer

The views expressed in this paper are those of the author(s) and do not necessarily reflect the views or policies of FAO.

## Conflict of Interest

The authors declare that the research was conducted in the absence of any commercial or financial relationships that could be construed as a potential conflict of interest. The reviewer EK declared a past co-authorship with one of the authors BM-L to the handling editor.

## Publisher's Note

All claims expressed in this article are solely those of the authors and do not necessarily represent those of their affiliated organizations, or those of the publisher, the editors and the reviewers. Any product that may be evaluated in this article, or claim that may be made by its manufacturer, is not guaranteed or endorsed by the publisher.

Frontiers Media SA remains neutral with regard to jurisdictional claims in published maps and institutional affiliations.

## Footnotes

^1^All references to Kosovo should be understood to be in the context of United Nations Security Council resolution 1244 (1999).
