# Supplementary material for: Descriptive and Multivariate Analysis of the Pig Sector in North Macedonia and Its Implications for African Swine Fever Transmission
Source: Front Vet Sci. 2021 Nov 30;8:733157. doi: 10.3389/fvets.2021.733157 (PMC8669509; doi:10.3389/fvets.2021.733157)
Supplement: Supplementary file 1 [file Data_Sheet_1.PDF]

ASF North Macedonia Survey

ASFNorthMacedonia

1: These questionnaires should be conducted by the interviewer, rather than just leaving it with the pig keeper. 2: It takes around 30 minutes for the pig keeper to reply to the questionnaire 3: When asking the questions, it is better to wait for the interviewed person to answer and then look for the answer in the list of options, rather than reading all the options to them, thus potentially misguiding their answers. 4: All questions refer to the period over the past 12 months, exclusively to what has happened over the past year. 5: Please turn on the GPS on your smartphone to capture location data

Contact Information

Name, LAST NAME of the interviewer

- ○ I am a placeholder answer

Name, LAST NAME of pig farmer

Village Name (where pigs are kept)

Municipality (Where pigs are kept)

- ○ VELLES
- ○ GRADSKO
- ○ DEMIR KAPIJA

Region name (where pigs are kept)

- ○ ISTOCHEN
- ○ PELAGONISKI
- ○ POLOSHKI

Date of Interview (dd/mm/yy)

Type of Farm

- ○ Commercial (big farm)
- ○ Family Farm
- ○ Backyard

Please Take a Picture of the Farm

Please Take the Location of the Farm

Enter the Farm's UIN Code

General Info and Socio-Economic Aspects

How many sows that had litters, do you have now?

What is the minimum number of sows that had litters over the past 12 months?

What is the maximum number of sows that had litters over the past 12 months?

How many boars do you have now?

What is the minimum number of boar over the past 12 months?

What is the maximum number of boars over the past 12 months?

How many fattened pigs do you have now?

What is the minimum number of fattened pigs over the past 12 months?

What is the maximum number of fattened pigs over the past 12 months?

How many piglets do you have now?

What is the minimum number of piglets eaten or sold over the past 12 months?

What is the maximum number of piglets eaten or sold over the past 12 months?

What breed of pigs do you keep?

- ☐ Local Breed
- ☐ Commercial breed (eg: Landrace, Large White, etc)

What percentage of your pigs are local breed?

What percentage of your pigs are commercial breed?

Are your pigs enclosed all year round? (Choose one of the following)

- ○ Yes, pigs are enclosed all year
- ○ No, the pigs are allowed to scavenge during the day, but return every night
- ○ No, the pigs scavenge for several days or months

How much of your household income comes from the pigs you raise(%)? (Write 0% if it is all for home consumption)

Who takes care of the pigs, i.e. feeding, cleaning the pen, etc? Choose from the list (more than one answer possible)

- ☐ Wife
- ☐ Husband
- ☐ Kids
- ☐ Other family members
- ☐ Hired personnel

If you have sows, when (over the past 12 months) did you get litters?

- ☐ Janurary
- ☐ February
- ☐ March
- ☐ April
- ☐ May
- ☐ June
- ☐ July
- ☐ August
- ☐ September
- ☐ October
- ☐ November
- ☐ December

Write the number of litters for the months indicated, in the following format: "Jan 20, Mar 50, Sep 20"

Health Status

How many pigs and piglets died on your farm or disappeared while free ranging, during last year?

How many pigs and piglets died of disease?

How many pigs and piglets disappeared/never returned during free ranging? (write 0 if none have disappeared)

Against which diseases did you vaccinate your pigs, over the past 12 month?

- ☐ I don't vaccinate
- ☐ Classical Swine Fever
- ☐ Erysipela
- ☐ Aujezsky
- ☐ Pasteurellosis
- ☐ Other (please specify)

If you selected Other (please specify)

How many times did you consult or interact with a veterinarian over the past 12 months (phones calls included)?

What did you do last time a pig was sick? Choose from the list (more than one answer possible)

- ☐ Separated the sick from the healthy ones

- ☐ Treated the animal/s yourself
- ☐ Consulted the veterinarian
- ☐ Slaughtered the sick pig for home consumption
- ☐ Slaughtered the sick pig and sold the meat
- ☐ Killed the sick pig and threw away the carcass
- ☐ Killed the sick pig and destroyed the carcass in my premises (by burial or burning)
- ☐ Killed the sick pig and destroyed the carcass outside my premises (by burial or burning)
- ☐ Sold the sick pig to a slaughterhouse
- ☐ Sold the sick pig to someone
- ☐ Sold the remaining healthy pigs (before they got sick) to a slaughterhouse
- ☐ Sold the remaining healthy pigs (before they got sick) to someone
- ☐ Slaughtered the remaining health pigs, before they got sick and kept the meat for home-consumption
- ☐ I cleaned and disinfected the pen(s)
- ☐ Did nothing

What did you do with the last adult pig that died? Choose from the list (more than one answer possible)

- ☐ Bury
- ☐ Throw away
- ☐ Dispose of in a pit
- ☐ Burn
- ☐ Fed to the pigs
- ☐ Fed to the dogs
- ☐ Consumed the meat

- [ ] Sold the meat
- [ ] Collected from household by dedicated services
- [ ] Contacted your private veterinarian
- [ ] Contacted veterinary authorities

Origin of Animals (Buying)

OPTIONAL: Origin of animals over past 12 months. This section is n ot applicable if all your animals were born at your farm or if you did not purchase any animal over the past 12 months. If there were no transactions do not fill in the form. If there were transactions, please fill in the form. Please capture all transactions over the past 12 months .

Where did you buy your pigs from (tick all that apply)?

- [ ] Commercial Farm
- [ ] Family Farm
- [ ] Backyard
- [ ] Middleman
- [ ] Live animal market

What type of pigs did you buy (tick all that apply)?

- [ ] Replacement Sows
- [ ] Boars
- [ ] Piglets for Fattening
- [ ] Pigs fattened half way

How many pigs did you buy in total?

When did you buy the pigs?

- [ ] January
- [ ] February
- [ ] March
- [ ] April
- [ ] May
- [ ] June
- [ ] July
- [ ] August
- [ ] September
- [ ] October
- [ ] November
- [ ] December

Please indicate all translations in the following format: "Farm type/Pig type/Number/Month/Village" Example 1: Commercial/Piglets for fattening/60/Jan/Village Example 2: Backyard/Boar/1/Apr/Village

Destination of Animals (Selling)

OPTIONAL: Destination of live animals (or whole carcasses) over the past 12 months. This section is not applicable if all your animals are slaughtered and consumed at your

household. If there were no transactions do not fill in the form. If there were transactions, please fill in the form. Please capture all transactions over the past 12 months .

Where did you sell your pigs to (tick all that apply)?

- [ ] Commercial Farm
- [ ] Family Farm
- [ ] Backyard
- [ ] Middleman
- [ ] Live animal market

What type of pigs did you sell (tick all that apply)?

- [ ] Ready-to-slaughter pigs
- [ ] Replacement sows
- [ ] Piglets for fattening
- [ ] Pigs fattened half way
- [ ] Boars

How many pigs did you sell in total?

[ ]

When did you sell the pigs (tick all that apply)?

- [ ] January
- [ ] February
- [ ] March
- [ ] April
- [ ] May
- [ ] June
- [ ] July

- ☐ August
- ☐ September
- ☐ October
- ☐ November
- ☐ December

Please indicate all translations in the following format: "Farm type/Pig type/Number/Month/Village" Example 1: Commercial/Replacement sows/10/Apr/Village Example 2: Backyard/Piglets for fattening/30/Apr/Village

Homeslaughter

How do you slaughter pigs? (choose one answer)

- ○ Slaughtered at home by household member
- ○ Slaughtered at home by someone else
- ○ Pigs are taken to slaughter (elsewhere) and I bring the meat back home
- ○ I do not consume the pigs I produce

In case of home slaughter, where do you get the equipment needed to slaughter and process the pig? (choose one)

- ○ We have all the equipment available at home
- ○ We have some of the equipment and some we barrow from our neighbors
- ○ We borrow all equipment from someone else

What do you do with inedible parts of the pig after homeslaughter? (more than one answer possible)

- ☐ Buried within premises

- [ ] Buried outside premises
- [ ] Burned within premises
- [ ] Burned outside premises
- [ ] Disposal in a pit
- [ ] Thrown away outside premises
- [ ] Disposal as household waste
- [ ] Collected by others (companies, municipality, etc.)
- [ ] Fed back to pigs
- [ ] Fed to dogs/cats
- [ ] Other (please specify)
- [ ] No nonedible parts left after slaughtering

If you selected other, please specify

When did you homeslaughter fattened pigs over the past 12 months?

- [ ] January
- [ ] February
- [ ] March
- [ ] April
- [ ] May
- [ ] June
- [ ] July
- [ ] August
- [ ] September
- [ ] October

- [ ] November
- [ ] December

Write the number of homeslaughter fattened pigs for the months indicated, in the following format: Jan 2, Mar 5, Sep 10

When did you homeslaughter piglets (under 3 months) over the past 12 months?

- [ ] January
- [ ] February
- [ ] March
- [ ] April
- [ ] May
- [ ] June
- [ ] July
- [ ] August
- [ ] September
- [ ] October
- [ ] November
- [ ] December

Write the number of homeslaughter piglets (under 3 months) for the months indicated, in the following format: Jan 2, Mar 5, Sep 10

What percentage of the homeslaughtered meat did you preserve (salt/smoke/dry)? (percentage)

How many months did it take your family to finish with all the preserved pork that you processed last season? (Number (in months))

Destination of products from homeslaughtered pigs (past 12 months)

What happens to the meat and products you produce? (Select all that apply)

- [ ] Home consumption
- [ ] Middleman
- [ ] Sold to restaurant/bar
- [ ] Sold to butcher/shop
- [ ] Sold/given to relatives, friends, neighbors

How much (in percentage) is consumed at home? (Write 0 if not applicable, answers have to add up to 100%)

How much (in percentage) is sold to the middleman? (Write 0 if not applicable, answers have to add up to 100%)

How much (in percentage) is sold to restaurant/bar? (Write 0 if not applicable, answers have to add up to 100%)

How much (in percentage) is sold to butcher/shop? (Write 0 if not applicable, answers have to add up to 100%)

How much (in percentage) is sold/given to relatives, friends, or neighbors? (Write 0 if not applicable, answers have to add up to 100%)

Where are your buyers located? (Select all that apply)

- ☐ Same village
- ☐ Same municipality
- ☐ Adjacent municipality
- ☐ Another region
- ☐ Skopje
- ☐ Neighboring country
- ☐ Any other country
- ☐ I do not know

What is the percentage of your buyers who are located in your village? (Write 0 if not applicable, answers have to add up to 100%)

What is the percentage of your buyers who are located in the same municipality? (Write 0 if not applicable, answers have to add up to 100%)

What is the percentage of your buyers who are located in an adjacent municipality? (Write 0 if not applicable, answers have to add up to 100%)

What is the percentage of your buyers who are located in another region? (Write 0 if not applicable, answers have to add

up to 100%)

What is the percentage of your buyers who are located in Skopje? (Write 0 if not applicable, answers have to add up to 100%)

What is the percentage of your buyers who are located in a neighboring country? (Write 0 if not applicable, answers have to add up to 100%)

What is the percentage of your buyers who are located in another country? (Write 0 if not applicable, answers have to add up to 100%)

If you are selling to another country, which one(s) is it?

What type of products do you sell or give away? (select all that apply)

- ☐ Sausage
- ☐ Fresh meat
- ☐ Fresh fat
- ☐ Dried/smoked/salted meat or fat
- ☐ Boiled/heat treated meat or fat

Biosecurity Basics and wild boar contact

Is your farm/home fenced?

- ○ Yes
- ○ No

Are your pigs kept in a pen or fenced area within your home?

- ○ Yes
- ○ No

Last time you bought pigs, how many days did you keep them isolated/separated from your pigs (quarantined)? Write 0 for

[ ]

Over the past 12 months, did you bring external boar into the farm to cross it with your sows?

- ○ Yes
- ○ The sows get crossed while outside my premises
- ○ No, I have my own boar
- ○ No, I have my own boar and also take him to other premises for breeding
- ○ No, I perform artificial insemination
- ○ No, there are no breeding animals (sows or boar) on the farm

Do you (or your workers) lend of borrow equipment to/from your neighbors?

- ○ Yes
- ○ No

Do you (or your workers) change shoes before going to the pigs?

- ○ Yes
- ○ No

Do you (or your workers) change clothes before going to the pigs?

- ○ Yes
- ○ No

Do you (or your workers) wash hands before going to the pigs?

- ○ Yes
- ○ No

Do you (or your workers) use a disinfection mat before going to the pigs?

- ○ Yes
- ○ No

Which persons are allowed to go to your pigs? (Select all that apply)

- ☐ Friends
- ☐ Neighbors
- ☐ Buyers
- ☐ Slaughterman
- ☐ Fellow pig farmers
- ☐ Veterinarians
- ☐ Nobody, access is restricted

When did you see wild boar close to your pigs over the past 12 months?

- ☐ Never
- ☐ January
- ☐ February
- ☐ March

- [ ] April
- [ ] May
- [ ] June
- [ ] July
- [ ] August
- [ ] September
- [ ] October
- [ ] November
- [ ] December

Do you hunt wild boar?

- ○ Yes
- ○ No

Biosecurity- Swill/scrap feeding

What do you feed your pigs?

- [ ] Grain/maize
- [ ] Commercial Feed
- [ ] Kitchen waste/food scraps
- [ ] Food processing by-products (e.g. from cheese processing, bakery, etc)
- [ ] Fresh grass
- [ ] Hay
- [ ] Slaughterhouse/Butcher leftovers
- [ ] Agricultural by-products

What is the percentage of grain/maize? (Write 0 if not applicable, answers have to add up to 100%)

What is the percentage of commercial feed? (Write 0 if not applicable, answers have to add up to 100%)

What is the percentage of kitchen waste/food scraps? (Write 0 if not applicable, answers have to add up to 100%)

What is the percentage of food processing by-products? (Write 0 if not applicable, answers have to add up to 100%)

What is the percentage fresh grass? (Write 0 if not applicable, answers have to add up to 100%)

What is the percentage of hay? (Write 0 if not applicable, answers have to add up to 100%)

What is the percentage of slaughterhouse/butcher leftovers? (Write 0 if not applicable, answers have to add up to 100%)

What is the percentage of agricultural by-products? (Write 0 if not applicable, answers have to add up to 100%)

If you feed kitchen waste/food scraps to your pigs, what is its origin?

- [ ] Own household
- [ ] Other households/neighbors
- [ ] Restaurant
- [ ] Market
- [ ] Other (Please specify)

If you selected other, please specify

If you feed kitchen waste/food scraps, do you boil them first?

- ○ Yes
- ○ No

Biosecurity Waste Management

What do you do with household waste (select all that apply)

- [ ] Collected by municipality
- [ ] Burned
- [ ] Buried within household premises
- [ ] Buried outside household premises
- [ ] Thrown within household premises
- [ ] Thrown outside household premises

Describe the way household waste is disposed in your village

- ○ No disposal site available
- ○ Fenced disposal site
- ○ Non-fenced disposal site
- ○ Buried at disposal site

- ○ Burned at disposal site

What months do you allow pigs to scavenge outside the household? (Select all that apply)

- ☐ Never
- ☐ January
- ☐ February
- ☐ March
- ☐ April
- ☐ May
- ☐ June
- ☐ July
- ☐ August
- ☐ September
- ☐ October
- ☐ November
- ☐ December

What do you do with the pig manure?

- ☐ Dump it outside my premises
- ☐ Store it (e.g. in a pit)
- ☐ Use it in fenced garden/field
- ☐ Use it in non-fenced garden/fields
- ☐ Sell it/give it to others

Biosecurity - Commercial farm and Family Farm

This form has to be filled in only in case of commercial and family farms!

Is your farm double fenced?

- ○ Yes
- ○ No

On your farm do you have established clean and dirty areas for your personal?

- ○ Yes
- ○ No

Do you apply detailed disinfection procedures to disinfect: vehicles, equipment and personnel entering your farm? Tick all that apply:

- ☐ Vehicles
- ☐ Equipment
- ☐ Personnel

Do you regulate what kind of food workers can bring to the farm?

- ○ Yes, certain products are not allowed
- ○ No, they can bring anything

Can workers on your farm keep pigs at home?

- ○ Yes
- ○ No

Can your workers go hunting in their free time?

- ○ Yes
- ○ No

How often do you organize events to raise the awareness and educate your workers / staff about ASF?

- ○ Never
- ○ Once a year
- ○ Twice a year
- ○ Every three months
- ○ Every month

How often do you evaluate the efficiency and enforcement of your biosecurity procedures?

- ○ Never
- ○ Once a year
- ○ Twice a year
- ○ Every three months
- ○ Each month

Awareness

Where do you get information regarding animal health? (Do not read out loud answers, Select all that apply)

- ☐ Radio
- ☐ TV
- ☐ Newspapers
- ☐ Veterinarian
- ☐ Local Authorities
- ☐ Rumors/Neighbors
- ☐ Leaflets/Posters
- ☐ Internet
- ☐ Church/ Mosque

Out of the following seven diseases that can affect pigs, select the ones that concern you the most? Choose three! (Read them all out loud. Tick the top three)

- [ ] Foot and mouth disease
- [ ] Swine influenza
- [ ] Seneca Valley Virus
- [ ] Classical swine fever
- [ ] Porcine Reproductive and Respiratory Syndrome (PRRS)
- [ ] African Swine Fever
- [ ] Aujeszky's disease

How did you hear about ASF? (Do not read the answers out loud, tick all that apply)

- [ ] I did not hear about it
- [ ] Radio
- [ ] TV
- [ ] Newspaper
- [ ] Veterinarian
- [ ] Local Authorities
- [ ] Rumors/Neighbors
- [ ] Leaflets/Posters
- [ ] Internet
- [ ] Church/ Mosque

Name three clinical signs that you think are related to ASF (read the answers out loud, tick all that apply)

- [ ] Fever
- [ ] Coughing
- [ ] Diarrhea

- ☐ Vomiting
- ☐ Reduced eating
- ☐ Joint swelling
- ☐ Hemorrhages in the skin
- ☐ Bloody diarrhea
- ☐ Bloody urine
- ☐ Sudden death
- ☐ I do not know any

Can ASF make humans sick?

- ○ Yes
- ○ No

Do you know how ASF can infect your pigs? (read the answers out loud, tick all that apply. If don't know, no answers are needed)

- ☐ Through badly produced vaccine
- ☐ Through the wind
- ☐ Bringing home infected animals
- ☐ Going to the pigs with infected boots and clothes
- ☐ Feeding infected pork products to the pigs
- ☐ mosquitoes
- ☐ Through the water from an infected river
- ☐ I don't know

Within what time frame would you report ASF if you suspected it on your farm? (Choose one)

- ○ I would wait a few days before reporting to avoid a false report
- ○ I would wait a few days before reporting to avoid financial losses

- ○ I would quickly report ASF, even if it could be a false alarm

What do you think is the reasons a pig owner would not report ASF? (Do not read the answer out loud, tick all that apply)

- ☐ Because people do not know how to report ASF
- ☐ Because it is too time consuming to report
- ☐ Because it is unclear what will happen after reporting
- ☐ Because selling pigs would be banned
- ☐ Because the pigs would be culled
- ☐ Because it would damage the reputation of those who reported
- ☐ Because there would be no compensation for the pigs that are culled
- ☐ Because pig owners prefer to deal with the disease themselves
