# Supplementary material for: Descriptive and Multivariate Analysis of the Pig Sector in North Macedonia and Its Implications for African Swine Fever Transmission
Source: Front Vet Sci. 2021 Nov 30;8:733157. doi: 10.3389/fvets.2021.733157 (PMC8669509; doi:10.3389/fvets.2021.733157)
Supplement: Supplementary file 2 [file Data_Sheet_2.PDF]

Appendix 2. African Swine Fever questionnaire responses summarized for multiple choice questions by proportion of respondents for North Macedonia and Kosovo between Sept 2019-Mar 2020. Proportion of respondents calculated as number of respondents selecting a given answer divided by number of respondents who answered a given question.

	Proportion Respondents (%)	
	**North Macedonia**	**Kosovo**
**General Info and Socio-Economic Aspects**		
**What breed of pigs do you keep?**		
Local Breed	25.8	96.0
Commercial breed (eg: Landrace, Large White, etc)	76.1	52.0
**Are your pigs enclosed all year round? (Choose one of the following)**		
Yes, pigs are enclosed all year	96.5	100.0
No, the pigs are allowed to scavenge during the day, but return every night	3.5	0.0
No, the pigs scavenge for several days or months	0.0	0.0
**Who takes care of the pigs, i.e. feeding, cleaning the pen, etc? Choose from the list (more than one answer possible)**		
Wife	48.1	80.0
Husband	79.6	100.0
Kids	21.0	44.0
Other family members	14.9	12.0
Hired personnel	7.9	4.0
**Health Status**		
**Against which diseases did you vaccinate your pigs, over the past 12 month?**		
I don't vaccinate	10.5	4.0
Classical Swine Fever	87.7	96.0
Erysipela	32.8	0.0
Aujezsky	2.6	4.0
Pasteurellosis	1.1	4.0
Other (please specify)	3.5	0.0
**What did you do last time a pig was sick? Choose from the list(more than one answer possible)**		
Separated the sick from the healthy ones	43.9	56.0
Treated the animal/s yourself	4.2	68.0
Consulted the veterinarian	85.4	84.0
Slaughtered the sick pig for home consumption	1.1	0.0
Slaughtered the sick pig and sold the meat	0.0	0.0
Killed the sick pig and threw away the carcass	0.2	0.0
Killed the sick pig and destroyed the carcass in my premises (by burial or burning)	1.8	0.0

Killed the sick pig and destroyed the carcass outside my premises (by burial or burning)	1.1	0.0
Sold the sick pig to a slaughterhouse	0.0	0.0
Sold the sick pig to someone	0.0	0.0
Sold the remaining healthy pigs (before they got sick) to a slaughterhouse	0.4	0.0
Sold the remaining healthy pigs (before they got sick) to someone	0.0	0.0
Slaughtered the remaining health pigs, before they got sick and kept the meat for home-consumption	0.4	0.0
I cleaned and disinfected the pen(s)	8.6	24.0
Did nothing	0.9	0.0
**What did you do with the last adult pig that died? Choose from the list (more than one answer possible)**		
Bury	47.3	8.0
Throw away	0.5	88.0
Dispose of in a pit	26.6	28.0
Burn	0.7	0.0
Fed to the pigs	0.0	0.0
Fed to the dogs	2.7	20.0
Consumed the meat	0.2	0.0
Sold the meat	0.0	0.0
Collected from household by dedicated services	3.0	0.0
Contacted your private veterinarian	19.7	4.0
Contacted veterinary authorities	12.7	0.0
**Origin of Animals (Buying)**		
**Where did you buy your pigs from (tick all that apply)?**		
Commercial Farm	8.8	44.0
Family Farm	3.3	56.0
Backyard	37.4	8.0
Middleman	0.0	28.0
Live animal market	8.3	24.0
**What type of pigs did you buy (tick all that apply)?**		
Replacement Sows	40.5	28.0
Boars	14.6	12.0
Piglets for Fattening	48.1	64.0
Pigs fattened half way	7.0	56.0
**When did you buy the pigs?**		
January	17.2	36.0
February	9.0	52.0
March	21.3	32.0
April	18.0	12.0
May	21.3	4.0
June	6.6	0.0

July	6.6	0.0
August	10.7	0.0
September	9.0	0.0
October	10.7	0.0
November	6.6	4.0
December	4.9	4.0
**Destination of Animals (Selling)**		
**Where did you sell your pigs to (tick all that apply)?**		
Commercial Farm	1.7	0.0
Family Farm	9.5	27.3
Backyard	49.3	81.8
Middleman	33.4	54.5
Live animal market	40.5	0.0
**What type of pigs did you sell (tick all that apply)?**		
Ready-to-slaughter pigs	50.9	63.6
Replacement sows	5.5	9.1
Piglets for fattening	69.4	54.5
Pigs fattened half way	11.4	45.5
Boars	0.7	0.0
**When did you sell the pigs (tick all that apply)?**		
January	25.4	0.0
February	20.6	0.0
March	25.8	9.1
April	21.8	27.3
May	21.4	36.4
June	18.1	27.3
July	17.7	0.0
August	18.5	9.5
September	24.6	4.0
October	36.7	54.5
November	58.1	45.5
December	42.3	9.1
**Homeslaughter**		
**How do you slaughter pigs? (choose one answer)**		
Slaughtered at home by household member	54.0	47.8
Slaughtered at home by someone else	22.1	52.2
Pigs are taken to slaughter (elsewhere) and I bring the meat back home	10.9	0.0
I do not consume the pigs I produce	12.9	0.0
**In case of home slaughter, where do you get the equipment needed to slaughter and process the pig? (choose one)**		
We have all the equipment available at home	62.9	39.1

We have some of the equipment and some we borrow from our neighbors	1.3	47.8
We borrow all equipment from someone else	0.8	13.0
Slaughterman has his own	35.1	
**What do you do with inedible parts of the pig after homeslaughter? (more than one answer possible)**		
Buried within premises	6.7	0.0
Buried outside premises	33.6	8.7
Burned within premises	2.0	0.0
Burned outside premises	0.7	0.0
Disposal in a pit	26.1	43.5
Thrown away outside premises	2.5	39.1
Disposal as household waste	0.0	0.0
Collected by others (companies, municipality, etc.)	2.2	4.3
Fed back to pigs	0.0	0.0
Fed to dogs/cats	16.4	95.7
Other (please specify)	0.2	0.0
No nonedible parts left after slaughtering	12.4	0.0
**When did you homeslaughter fattened pigs over the past 12months?**		
January	9.8	0.0
February	3.5	0.0
March	5.2	0.0
April	2.9	0.0
May	2.3	0.0
June	1.4	0.0
July	1.7	0.0
August	1.7	0.0
September	4.6	0.0
October	13.0	25.0
November	60.8	100.0
December	27.1	50.0
**When did you homeslaughter piglets (under 3 months) over the past 12 months?**		
January	10.3	0.0
February	3.4	0.0
March	4.4	0.0
April	8.9	0.0
May	31.0	18.2
June	13.3	77.3
July	2.0	45.5
August	6.4	4.5
September	5.4	0.0
October	7.9	0.0

November	21.7	0.0
December	17.2	0.0
**Destination of products from homeslaughtered pigs (past 12months)**		
**What happens to the meat and products you produce? (Select all that apply)**		
Home consumption	90.2	100.0
Middleman	5.0	32.0
Sold to restaurant/bar	0.3	16.0
Sold to butcher/shop	8.2	0.0
Sold/given to relatives, friends, neighbors	5.8	80.0
**Where are your buyers located? (Select all that apply)**		
Same village	40.5	100.0
Same municipality	46.7	95.2
Adjacent municipality	24.1	52.4
Another region	7.7	19.0
Skopje	8.7	28.6
Neighboring country	0.0	0.0
Any other country	0.0	0.0
I do not know	19.5	0.0
**What type of products do you sell or give away? (select all that apply)**		
Sausage	43.5	14.3
Fresh meat	87.9	100.0
Fresh fat	21.8	38.1
Dried/smoked/salted meat or fat	31.5	81.0
Boiled/heat treated meat or fat	3.2	0.0
**Biosecurity Basics and wild boar contact**		
**Is your farm/home fenced?**		
Yes	90.6	100.0
No	9.4	0.0
**Are your pigs kept in a pen or fenced area within your home?**		
Yes	98.2	92.0
No	1.8	8.0
**Over the past 12 months, did you bring external boar into the farm to cross it with your sows?**		
Yes	8.6	32.0
The sows get crossed while outside my premises	2.9	12.0
No, I have my own boar	35.9	12.0
No, I have my own boar and also take him to other premises for breeding	0.4	0.0
No, I perform artificial insemination	35.9	4.0
No, there are no breeding animals (sows or boar) on the farm	16.2	40.0

**Do you (or your workers) lend of borrow equipment to/from your neighbors?**		
Yes	3.7	72.0
No	96.3	28.0
**Do you (or your workers) change shoes before going to the pigs?**		
Yes	94.1	80.0
No	5.9	20.0
**Do you (or your workers) change clothes before going to the pigs?**		
Yes	92.8	40.0
No	7.2	60.0
**Do you (or your workers) wash hands before going to the pigs?**		
Yes	87.1	28.0
No	12.9	72.0
**Do you (or your workers) use a disinfection mat before going to the pigs?**		
Yes	68.5	4.0
No	31.5	96.0
**Which persons are allowed to go to your pigs? (Select all that apply)**		
Friends	9.0	0.0
Neighbors	8.5	28.0
Buyers	8.1	36.0
Slaughterman	4.2	28.0
Fellow pig farmers	1.8	76.0
Veterinarians	86.7	100.0
Nobody, access is restricted	23.6	0.0
**When did you see wild boar close to your pigs over the past 12months?**		
Never	91.2	96.0
January	0.4	0.0
February	0.7	0.0
March	0.2	0.0
April	0.2	0.0
May	0.2	0.0
June	0.0	0.0
July	0.2	0.0
August	0.4	0.0
September	0.4	0.0
October	1.3	0.0
November	1.3	0.0
December	1.3	4.0
**Do you hunt wild boar?**		
Yes	2.4	8.0
No	97.6	92.0

Biosecurity- Swill/scrap feeding		
**What do you feed your pigs?**		
Grain/maize	97.2	100.0
Commercial Feed	38.7	44.0
Kitchen waste/food scraps	6.8	80.0
Food processing by-products (e.g. from cheese processing, bakery, etc)	0.7	56.0
Fresh grass	15.1	60.0
Hay	7.2	84.0
Slaughterhouse/Butcher leftovers	0.2	0.0
Agricultural by-products	6.6	8.0
**If you feed kitchen waste/food scraps to your pigs, what is its origin?**		
No swill	89.0	12.0
Own household	10.5	88.0
Other households/neighbors	0.0	4.0
Restaurant	0.2	0.0
Market	0.4	0.0
Other (Please specify)	0.0	0.0
**If you feed kitchen waste/food scraps, do you boil them first?**		
No swill	88.8	12.0
Yes	6.3	0.0
No	4.8	88.0
**Biosecurity Waste Management**		
**What do you do with household waste (select all that apply)**		
Collected by municipality	77.2	68.0
Burned	9.2	4.0
Buried within household premises	3.1	0.0
Buried outside household premises	0.0	0.0
Thrown within household premises	0.0	0.0
Thrown outside household premises	8.1	36.0
**Describe the way household waste is disposed in your village**		
No disposal site available	33.8	80.0
Fenced disposal site	46.8	12.0
Non-fenced disposal site	11.1	4.0
Buried at disposal site	2.5	4.0
Burned at disposal site	5.8	0.0
**What months do you allow pigs to scavenge outside the household? (Select all that apply)**		
Never	99.3	100.0
January	0.0	0.0
February	0.0	0.0
March	0.0	0.0
April	0.0	0.0

May	0.0	0.0
June	0.0	0.0
July	0.0	0.0
August	0.0	0.0
September	0.2	0.0
October	0.7	0.0
November	0.7	0.0
December	0.0	0.0
**What do you do with the pig manure?**		
Dump it outside my premises	8.3	84.0
Store it (e.g. in a pit)	36.5	20.0
Use it in fenced garden/field	27.8	8.0
Use it in non-fenced garden/fields	49.2	36.0
Sell it/give it to others	1.3	32.0
**Biosecurity - Commercial farm and Family Farm**		
**Is your farm double fenced?**		
Yes	39.2	40.0
No	60.8	60.0
**On your farm do you have established clean and dirty areas for your personal?**		
Yes	55.5	25.0
No	45.5	75.0
**Do you apply detailed disinfection procedures to disinfect: vehicles, equipment and personnel entering your farm? Tick all that apply:**		
Vehicles	55.3	33.3
Equipment	68.8	100.0
Personnel	65.2	33.3
**Do you regulate what kind of food workers can bring to the farm?**		
Yes, certain products are not allowed	42.1	25.0
No, they can bring anything	57.9	75.0
**Can workers on your farm keep pigs at home?**		
Yes	13.2	25.0
No	86.8	75.0
**Can your workers go hunting in their free time?**		
Yes	8.8	25.0
No	91.2	75.0
**How often do you organize events to raise the awareness and educate your workers / staff about ASF?**		
Never	43.1	75.0
Once a year	14.6	25.0
Twice a year	15.4	0.0
Every three months	12.3	0.0
Every month	14.6	0.0

**How often do you evaluate the efficiency and enforcement of your biosecurity procedures?**		
Never	31.8	75.0
Once a year	11.6	0.0
Twice a year	10.9	25.0
Every three months	18.6	0.0
Every month	27.1	0.0
**Awareness**		
**Where do you get information regarding animal health? (Do not read out loud answers, Select all that apply)**		
Radio	20.8	4.0
TV	75.6	72.0
Newspapers	18.2	32.0
Veterinarian	96.3	96.0
Local Authorities	4.4	48.0
Rumors/Neighbors	9.0	4.0
Leaflets/Posters	29.8	20.0
Internet	39.4	12.0
Church/ Mosque	0.0	0.0
**Out of the following seven diseases that can affect pigs, select the ones that concern you the most? Choose three! (Read them all out loud. Tick the top three)**		
Foot and mouth disease	16.2	68.0
Swine influenza	41.4	92.0
Seneca Valley Virus	0.7	0.0
Classical swine fever	85.3	92.0
Porcine Reproductive and Respiratory Syndrome (PRRS)	19.0	4.0
African Swine Fever	85.6	32.0
Aujeszky's disease	0.0	4.0
**How did you hear about ASF? (Do not read the answers out loud, tick all that apply)**		
I did not hear about it	1.5	32.0
Radio	26.3	0.0
TV	84.5	28.0
Newspapers	19.3	8.0
Veterinarian	90.8	60.0
Local Authorities	2.8	24.0
Rumors/Neighbors	7.9	4.0
Leaflets/Posters	25.8	4.0
Internet	30.2	4.0
Church/ Mosque	0.0	0.0
**Name three clinical signs that you think are related to ASF (read the answers out loud, tick all that apply)**		

Fever	60.0	68.0
Coughing	13.6	16.0
Diarrhea	35.0	64.0
Vomiting	18.2	0.0
Reduced eating	60.0	44.0
Joint swelling	10.9	0.0
Hemorrhages in the skin	60.6	12.0
Bloody diarrhea	18.8	16.0
Bloody urine	0.9	0.0
Sudden death	52.1	40.0
I do not know any	2.4	24.0
**Can ASF make humans sick?**		
Yes	1.5	0.0
No	98.5	100.0
**Do you know how ASF can infect your pigs? (read the answers out loud, tick all that apply. If don't know, no answers are needed)**		
Through badly produced vaccine	1.8	0.0
Through the wind	3.5	0.0
Bringing home infected animals	87.1	76.0
Going to the pigs with infected boots and clothes	49.9	20.0
Feeding infected pork products to the pigs	39.2	28.0
mosquitoes	20.4	0.0
Through the water from an infected river	14.2	0.0
I don't know	0.0	20.0
**Within what time frame would you report ASF if you suspected it on your farm? (Choose one)**		
I would wait a few days before reporting to avoid a false report	23.2	12.0
I would wait a few days before reporting to avoid financial losses	0.4	40.0
I would quickly report ASF, even if it could be a false alarm	76.4	48.0
**What do you think is the reasons a pig owner would not report ASF? (Do not read the answer out loud, tick all that apply)**		
Because people do not know how to report ASF	39.6	60.0
Because it is too time consuming to report	0.9	28.0
Because it is unclear what will happen after reporting	31.1	64.0
Because selling pigs would be banned	24.3	36.0
Because the pigs would be culled	27.8	16.0
Because it would damage the reputation of those who reported	15.1	20.0
Because there would be no compensation for the pigs that are culled	9.8	0.0
Because pig owners prefer to deal with the disease themselves	2.4	0.0
