# Supplementary material for: Descriptive and Multivariate Analysis of the Pig Sector in North Macedonia and Its Implications for African Swine Fever Transmission
Source: Front Vet Sci. 2021 Nov 30;8:733157. doi: 10.3389/fvets.2021.733157 (PMC8669509; doi:10.3389/fvets.2021.733157)
Supplement: Supplementary file 3 [file Table_1.pdf]

Supplementary Table 1. Questions and attributed score by answer used to generate biosecurity risk scores for North Macedonian pig producers administered questionnaires between September 2019-March 2020.

All Farms	
**Question**	
**Answer**	**Attributed Score**
**Are pigs enclosed all year round?**	
Yes, the pigs are enclosed all year	0
No, the pigs are allowed to scavenge during the day, but return every night<br>No, the pigs scavenge for several days or months	1
Against what diseases did you vaccinate your pigs, over the past 12 month?	
Classical Swine Fever<br>Erysipela<br>Aujezsky's<br>Pasteurellosis<br>Other	0
I don't vaccinate	1
What did you do the last time a pig got sick?	
Separated the sick from the healthy ones<br>Treated the animal/s yourself<br>Consulted the veterinarian<br>Slaughtered the sick pig for home consumption<br>Slaughtered the sick pig and sold the meat<br>Killed the sick pig and threw away the carcass<br>Killed the sick pig and destroyed the carcass in my premises (by burial or burning)<br>Killed the sick pig and destroyed the carcass outside my premises (by burial or burning)<br>Slaughtered the remaining healthy pigs<br>I cleaned and disinfected the pen(s)	0
Sold the sick pig to a slaughterhouse<br>Sold the sick pig to someone<br>Sold the remaining healthy pigs (before the became sick) to a slaughterhouse<br>Sold the remaining healthy pigs (before the became sick) to someone<br>Did nothing	1
What did you do with the last adult pig that died?	
Bury<br>Throw away<br>Dispose of in a pit	0

Burn<br>Fed to the dogs<br>Consumed the meat<br>Collected from household by dedicated services<br>Contacted your private veterinarian<br>Contacted veterinary authorities	
Sold the meat<br>Fed to the pigs	1
**What did you do with the inedible parts of the pig after homeslaughter?**	
Buried within premises<br>Buried outside premises<br>Burned within premises<br>Burned outside premises<br>Disposal in a pit<br>Disposal as household waste<br>Collected by others (companies, municipality, etc.)<br>Fed to dogs/cats<br>Other<br>No nonedible parts left after slaughtering	0
Fed back to pigs<br>Thrown away outside premises	1
**Is your farm/home fenced?**	
Yes	0
No	1
**Are your pigs kept in a pen or fenced area within your home?**	
Yes	0
No	1
**Last time you bought pigs, how many days did you keep them isolated/separated from your pigs (quarantined)? Write 0 for "no quarantine". Question converted to quarantines new pigs yes/no. Answer 0=no, >0=yes**	
Yes	0
No	1
**Over the past 12 months, did you bring external boar into the farm to cross it with your sows?**	
No, I have my own boar<br>No, I perform artificial insemination<br>No, there are no breeding animals (sows or boar) on the farm	0
Yes<br>No, I have my own boar and also take him to other premises for breeding<br>The sows get crossed while outside my premises	1
**Do you (or your workers) lend or borrow equipment to/from your neighbors?**	
No	0
Yes	1

Do you (or your workers) change shoes before going to the pigs?	
Yes	0
No	1
Do you (or your workers) change clothes before going to the pigs?	
Yes	0
No	1
Do you (or your workers) wash hands before going to the pigs?	
Yes	0
No	1
Do you (or your workers) use a disinfection mat before going to the pigs?	
Yes	0
No	1
Which persons are allowed to go to your pigs?	
Nobody, access is restricted	0
Friends<br>Neighbors<br>Buyers<br>Slaughterman<br>Fellow pig farmers<br>Veterinarians	1
When did you see wild boar close your pigs over the past 12 months? Converted to yes/no: never=no, any other selection=yes	
Never	0
January<br>February<br>March<br>April<br>May<br>June<br>July<br>August<br>September<br>October<br>November<br>December	1
Do you hunt wild boar?	
No	0
Yes	1
What do you feed your pigs?	
Grain/maize<br>Commercial feed<br>Food processing-by-products (e.g. from cheese processing, bakery, etc.)	0

Fresh grass<br>Hay<br>Agricultural by-products	
Kitchen waste/food scraps<br>Slaughterhouse/Butcher leftovers	1
If you feed kitchen waste/food scraps, do you boil them first?	
Yes	0
No	1
What months do you allow pigs to scavenge outside the household? Converted to yes/no: never=no, any other answer=yes	
Never	0
January<br>February<br>March<br>April<br>May<br>June<br>July<br>August<br>September<br>October<br>November<br>December	1
Name three clinical signs that you think are related to ASF (read the answers out loud and tick all the apply)	
Fever<br>Coughing<br>Diarrhea<br>Vomiting<br>Reduced eating<br>Joint swelling<br>Hemorrhages in the skin<br>Bloody diarrhea<br>Bloody urine<br>Sudden death	0
I don't know	1

Family and Commercial Farms

**Question**	
**Answer**	**Attributed Score**
Is your farm double fenced?	

Yes	0
No	1
On your farm do you have established clean and dirty areas for your personnel?	
Yes	0
No	1
Do you regulate what kind of food workers can bring to the farm?	
Yes, certain products are not allowed	0
No, they can bring anything	1
Can workers on your farm keep pigs at home?	
No	0
Yes	1
Can your workers go hunting in their free time?	
No	0
Yes	1
How often do you organize events to raise the awareness and educate your workers / staff about ASF?	
Once a year<br>Twice a year<br>Every three months<br>Every month	0
Never	1
How often do you evaluate the efficiency and enforcement of your biosecurity procedures?	
Once a year<br>Twice a year<br>Every three months<br>Each month	0
Never	1
